# GluN2B-mediated regulation of silent synapses for receptor specification and addiction memory

**DOI:** 10.1038/s12276-025-01399-z

**Published:** 2025-02-10

**Authors:** Hyun Jin Kim, Sangjun Lee, Gyu Hyun Kim, Kibong Sung, Taesik Yoo, Jung Hyun Pyo, Hee-Jung Jo, Sanghyeon Lee, Hyun-Young Lee, Jung Hoon Jung, Kea Joo Lee, Joung-Hun Kim

**Affiliations:** 1 Department of Life Sciences, Pohang University of Science and Technology, Pohang, Republic of Korea; 2 Salk Institute for Biological Studies, La Jolla, CA USA; 3 Department of Biomedical Science and Engineering, Gwangju Institute of Science and Technology, Gwangju, Republic of Korea; 4 Neural Circuits Research Group, Korea Brain Research Institute, Daegu, Republic of Korea; 5 Department of Neuroscience, Korea University College of Medicine, Seoul, Republic of Korea; 6 Department of Biomedical Sciences, Seoul National University College of Medicine, Seoul, Republic of Korea; 7 College of Pharmacy, Keimyung University, Daegu, Republic of Korea

**Keywords:** Synaptic plasticity, Molecular neuroscience, Synaptic transmission

## Abstract

Psychostimulants, including cocaine, elicit stereotyped, addictive behaviors. The reemergence of silent synapses containing only NMDA-type glutamate receptors is a critical mediator of addiction memory and seeking behaviors. Despite the predominant abundance of GluN2B-containing NMDA-type glutamate receptors in silent synapses, their operational mechanisms are not fully understood. Here, using conditional depletion/deletion of GluN2B in D1-expressing accumbal medium spiny neurons, we examined the synaptic and behavioral actions that silent synapses incur after repeated exposure to cocaine. GluN2B ablation reduces the proportion of silent synapses, but some of them can persist by substitution with GluN2C, which drives the aberrantly facilitated synaptic incorporation of calcium-impermeable AMPA-type glutamate receptors (AMPARs). The resulting precocious maturation of silent synapses impairs addiction memory but increases locomotor activity, both of which can be normalized by the blockade of calcium-impermeable AMPAR trafficking. Collectively, GluN2B supports the competence of cocaine-induced silent synapses to specify the subunit composition of AMPARs and thereby the expression of addiction memory and related behaviors.

## GluN2B role in cocaine addiction memory revealed

Cocaine creates ‘silent synapses’, immature brain connections lacking AMPA receptors. Researchers studied mice with GluN2B, a key protein in these synapses, which was selectively removed from specific brain cells. Using genetic tools and electrophysiology, they found that GluN2B removal reduced silent synapses and weakened drug-related memory but unexpectedly increased drug sensitivity. The findings suggest that the removal of GluN2B leads to fewer silent synapses but GluN2B aids in their maturation by adding calcium-insensitive AMPA receptors. This highlights GluN2B’s crucial role in maintaining silent synapses, providing new insights into addiction memory and potential treatment strategies.

This summary was initially drafted using artificial intelligence, then revised and fact-checked by the author.

## Introduction

Neural circuits and the physiological mechanisms underlying addiction memory and the associated behaviors in response to psychostimulants such as cocaine have been extensively explored^1^. For example, the mesocorticolimbic system is regulated by dopamine, which can elaborate and modulate synaptic plasticity, modulating the acquisition and maintenance of addiction memory^2–5^. A substantial portion of dopaminergic projections originating from the ventral tegmental area extends to the striatum, where they innervate medium spiny neurons (MSNs) expressing either dopamine receptor D1 (Drd1) or D2 (Drd2)^6^. The dopamine-dependent regulation of synapses in MSNs of the nucleus accumbens (NAc) is believed to adjust or determine the development and intensification of addiction memory and seeking behaviors toward cocaine^7^.

Neural innervations from the basolateral amygdala (BLA) to the NAc undergo synaptic plasticity, leading to motivated behaviors toward cocaine, as well as cue-induced reinstatement, for example, a selective enhancement of transmission strength from the BLA to Drd1-expressing MSNs (D1-MSNs) after chronic cocaine exposure^8,9^. Consistently, stimulation of the BLA–NAc pathway alone amplifies the magnitude of addiction-related behaviors, including locomotor sensitization^8,10^ and conditioned place preference (CPP)^11^, whereas the activation of cortical projections does not^12^. Furthermore, the cell-type-specific output activity of NAc D1-MSNs exerts bidirectional effects on the extinction and reinstatement of cocaine seeking, along with CPP being disrupted by the modulation of actin polymerization within the NAc shell (NAcSh)^13,14^. Hence, various forms of plasticity occurring at each type of MSN seem to differentially modulate the behavioral changes triggered by cocaine administration.

The generation and maturation of silent synapses lacking α-amino-3-hydroxy-5-methyl-4-isoxazolepropionic acid glutamate receptors (AMPARs)^15^ in the NAc could be major mediators of the development and intensification of cocaine-induced behaviors^15–17^. While silent synapses containing only *N*-methyl d-aspartate glutamate receptors (NMDARs) typically occur in the early stages of neuronal development but diminish when neurons mature, they reappear after exposure to addictive substances, including cocaine or amphetamine, which might represent a ‘rejuvenation’ of neurons^17^.

Cocaine-generated silent synapses are preferentially composed of GluN2B-containing NMDARs (GluN2B-NMDARs) and undergo maturation with the incorporation of AMPARs^18^, which can support the expression of addiction memory and seeking behaviors^15,19^. Despite their mechanistic and clinical importance, whether only GluN2B-NMDARs are able to constitute and maintain silent synapses, how they modulate synaptic modification and which physiological significance they hold in the context of drug addiction remained to be clarified, particularly at the circuit level. Using a combination of region- and cell-type-specific depletion/deletion of GluN2B, electrophysiological recordings, electron and confocal microscopy analyses, and behavioral tests, we characterized the operational roles of GluN2B-NMDARs in cocaine-induced silent synapses in NAc D1-MSNs and the physiological and behavioral roles of GluN2B-NMDARs after the repeated administration of cocaine.

## Methods

### Experimental animals

All procedures followed the animal care guidelines approved by the POSTECH Institutional Animal Care and Use Committee (Pohang University of Science and Technology, POSTECH-2019-0060). D1a-Cre mice were purchased from Mutant Mouse Resource and Research Centers (MMRRC) (#034258-UCD), and Rosa26-LSL-Cas9 knock-in (#024857) mice were obtained from Jackson Laboratory. The mice were housed in temperature- and humidity-controlled rooms with a light/dark (12 h/12 h) cycle and ad libitum access to food and water.

### DNA constructs

We used a suite of short hairpin RNA (shRNA) design tools, including BLOCK-It RNAi Designer, siDESIGN Center and AsiDesigner, for the production of shRNAs targeting the mouse GluN2B sequence GCTCATCTCTGTGTCATAT. Once designed, the shRNAs were confirmed and incorporated into the pAAV-Ef1a-DIO-cKD-eYFP-Blank construct, as detailed in earlier studies^9,20^. We expressed both photoactive (PA) and photoinsensitive (PI) Rac1 by cloning the PA-Rac1 (Addgene plasmid 22027) and PI-Rac1 (Addgene plasmid 22028) constructs into pAAV-EF1a-double floxed-hChR2(H134R)-eYFP-WPRE (Addgene plasmid 20298)^21^.

A target sequence including the PAM site (CGGGACTGTATTCCGCATGCAGG–AGG) of sgGrin2b was identified using online search tools to express sgGrin2b. The oligomer with this sequence was then integrated into the pSMART vector (Addgene plasmid 80427) to drive single guide RNA (sgRNA) transcription from the U6 promoter^22^. The resulting U6-sgGrin2B-scaffold constructs were subsequently cloned and inserted into the MluI site of the pAAV-EF1a-DIO-mCherry vector (Addgene plasmid 20299).

### Virus production

Virus production was conducted in accordance with established protocols^23^. In brief, HEK-293 cells were cotransfected with helper plasmids (delta-F6), capsid (serotype 5) and pAAV vectors (pAAV-Ef1a-DIO-cKD-eYFP-shGluN2B, pAAV-Ef1a-DIO-cKD-eYFP-Blank, pAAV-Ef1a-DIO-PA-Rac1-mCherry, pAAV-Ef1a-DIO-PI-Rac1-mCherry, pAAV-U6-sgGrin2b-Ef1a-DIO-mCherry and pAAV-U6-scrambled-Ef1a-DIO-mCherry) at an equal molar ratio using LipoFector-2000 transfection reagents (AB-LF-2002, AptaBio). Seventy-two hours after cotransfection, the cells were lysed through freeze‒thaw cycles, and the resulting AAV particles were concentrated with an Amicon filter (100 kDa, Millipore) to achieve at least 3.0 × 10^12^ genome copies per milliliter.

### Animal surgery

The mice were anesthetized using ketamine and xylazine before undergoing surgery using a stereotaxic apparatus (Kopf). A 0.3–0.5 μl volume of the virus solution was microinfused with Nanoject II or III (Drummond Scientific Instrument) for 1 min (23.0 nl per injection, rate of 46 nl/s), followed by a 10-min pause for virus diffusion. The coordinates were designated according to the mouse brain atlas (NAcSh: Anterior-Posterior (AP) +1.35 mm, Medial-Lateral (ML) ±0.5 mm, Dorsal-Ventral (DV) −4.50 mm from the bregma; BLA: AP −1.40 mm, ML ±3.35, DV −4.50 mm from the bregma). For the microinfusion of drugs or peptides, bilateral guide cannulas (26-gauge, center-to-center distance: 0.5 mm, Plastics One) were implanted 0.5 mm above the injection sites and fixed on the skull with screws and dental cement. The injection cannula remained capped with a dummy cannula and dust cap (Plastics One) after surgery. For in vivo optical illumination, optical fibers (numerical aperture 0.37, fiber diameter 200 μm, ferrule diameter φ2.5 mm, fiber length 5.0 mm; Newdoon) were implanted at a 15° angle with the following coordinates: AP +1.30 mm, ML ±1.5 mm, and DV −4.53 mm.

### Behavioral analyses

CPP was performed one day after 5 days of cocaine injections, as described previously^24^. The CPP chambers were composed of white or black-lined acrylic boxes (20 × 20 cm) connected with a narrow gray corridor (Supplementary Fig. 1i). Except during acquisition, the mice were always placed into the connecting chamber. During acclimation, after a sham intraperitoneal (i.p.) injection, the mice explored the CPP chambers for 15 min. Before the beginning of the test sessions, a preference test was performed to determine which chamber would be paired with cocaine or saline (pretest). The preferred place was assigned as paired with the saline injection, whereas the less preferred place was paired with the cocaine injection. If the preference was unclear (time difference <30 s), the white box was paired with cocaine and the black box was paired with saline. For sucrose CPP, the mice were subjected to water deprivation for 3 h before the conditioning sessions. When the conditioning session started, Falcon tube lids containing either a 15% sucrose solution or plain water were placed. The animals were then allowed to explore and consume the solution for 15 min. The CPP score equaled the difference in time spent between the two chambers. Movement paths and durations were analyzed using SMART 3.0 software (PanLab), with the position of each mouse determined by its front paw location.

Cocaine-induced locomotion was measured in a 40 × 60 cm white box, and the mice were subjected to two 10-min sessions where they explored freely and acclimatized. The next day, after an i.p. injection of saline to establish basal activity, the mice were injected with either saline (0.9%) or cocaine (20 mg/kg) in their home cages over five consecutive days. On each treatment day, after the injection, the mice were placed at the center of the box, and their movements were monitored for 10 min after a 1-min acclimation period. The movement trajectories were analyzed using SMART 2.0 and 3.0 software (PanLab). For the photoilluminated locomotor and CPP experiments (Fig. 5b, e, f and Supplementary Fig. 5f), light was shone after the cocaine injection during the open field or CPP tests. The optical fibers were connected to a 473-nm blue laser diode (ThorLabs), which remained illuminated throughout the behavioral sessions with an output of 0.35 mW.

### Electrophysiology

Acute brain slices were prepared for electrophysiological recordings within 3 h after the last injection of cocaine. As described previously^9^, coronal NAc slices (300 μm thick) were obtained using a vibratome (VT1200s, Leica) in an ice-cold sucrose cutting solution containing (in mM) 20 NaCl, 3.5 KCl, 1.4 NaH_2_PO_4_, 1.3 MgCl_2_, 26 NaHCO_3_, 11 d-glucose and 175 sucrose while it was equilibrated with 95% O_2_/5% CO_2_ (pH 7.3–7.4). The obtained slices were maintained in artificial cerebrospinal fluid (aCSF) containing (in mM) 126 NaCl, 18 NaHCO_3_, 11 d-glucose, 1.6 KCl, 1.2 NaH_2_PO_4_, 1.2 MgCl_2_ and 2.5 CaCl_2_ and were constantly bubbled with 95% O_2_/5% CO_2_ (~295–305 mOsm, pH 7.3–7.4) at room temperature after 30 min of recovery at 33 °C. Slices were placed in recording chambers and continuously perfused (2 ml/min) with aCSF at room temperature to avoid a temperature-dependent increase in NMDAR excitatory postsynaptic currents (EPSCs)^5^, and then a whole-cell voltage‒clamp configuration was established with a MultiClamp 700B amplifier (Molecular Devices). Recording electrodes (5–7 MΩ; Narishige) were filled with an internal solution containing (in mM) 117 CsMeSO_4_, 20 HEPES, 0.4 EGTA, 2.8 NaCl, 5 TEA-Cl, 2.5 MgATP, 0.25 Na_3_GTP and 5 QX-314 (pH 7.2 and 275–285 mOsm adjusted with CsOH and HEPES, respectively). Recordings were conducted under visual guidance (40×, differential interference contrast optics), and the expression of eYFP for verification of D1-MNNs was confirmed before the recordings. A series resistance of 10–30 MΩ was monitored continuously throughout the recordings. Upon observing changes greater than 15%, any recorded data were excluded from further analysis. Synaptic currents were recorded, filtered at 3 kHz, amplified five times and then digitized at 20 kHz with a Digidata 1322A analog-to-digital converter (Molecular Devices).

We applied various chemicals at various concentrations as follows: picrotoxin (PTX, 100 μM; Tocris) for the blockade of GABAaR-mediated responses; tetrodotoxin (TTX, 1 μM; Tocris) for the blockade of sodium channel-mediated action potentials; and 2-amino-5-phosphonovaleric acid (APV, 50 μM; Tocris) for the blockade of NMDAR-mediated responses. Ifenprodil hemitartrate (3 μM, 0545, Tocris) and (2S*,3R*)-1-(phenanthrene-2-carbonyl) piperazine-2,3-dicarboxylic acid (PPDA; 0.1 μM, 2530, Tocris) were used to inhibit GluN2B- and GluN2C/D-containing NMDARs, respectively. 2,3-Dihydroxy-6-nitro-7-sulfamoyl-benzo[F]quinoxaline (NBQX; 25 μM, 0373, Tocris) was used to inhibit AMPAR-mediated responses. 1-Napthylacetyl spermine trihydrochloride (NASPM; 200 μM, 2766, Tocris) was used to selectively inhibit GluA2-lacking AMPARs.

Optical stimulation of the axonal fibers of BLA pyramidal neurons to evoke EPSCs was achieved via illumination with 0.5-msec blue light pulses from a 470-nm light emitting diode source (M470L3, ThorLabs). Optically evoked AMPAR- and NMDAR-EPSCs were measured at −70 mV in the presence of APV and at +50 mV in the presence of NBQX, respectively. With 10–30 consecutive individual AMPAR-EPSCs, a nonstationary fluctuation analysis (NSFA) was performed with MiniAnalysis (Synaptosoft). The decay kinetics of NMDAR-EPSCs were assessed using the time from the peak amplitudes of EPSCs to the one-half peak amplitudes^6,7^. The mean amplitude of NMDAR-EPSCs was measured by averaging 10–20 consecutives individual EPSCs at various holding potentials (−80, −50, −30, 0, 30 and 50 mV) in the presence of NBQX and PTX. The antagonist sensitivity of NMDAR-EPSCs was measured by subtracting the peak value of the mean amplitude recorded 10 min after each antagonist treatment from the mean peak amplitude before treatment. The resulting values were then divided by the mean peak amplitudes before treatment (the ifenprodil treatment was followed by a PPDA perfusion 15 min later). Miniature EPSCs (mEPSCs) were recorded at a −70 mV holding potential in the presence of TTX and PTX for 3 min. mEPSCs were detected using a template-based event-detecting module from Clampfit 10.7 software (Molecular Devices).

We used the minimal stimulation method to estimate the proportion of silent synapses, as previously described^5,15^. The frequency of optical stimulation was set at 0.33 Hz. After EPSCs (<50 pA) were evoked at −70 mV, the photostimulation intensity was adjusted until both failure and success could be readily distinguished. The proportion of silent synapses was calculated using the following equation: 1 − ln(*F*_−70_)/ln(*F*_+50_), where *F*_−70_ is the failure rate at −70 mV and *F*_+50_ is the failure rate at +50 mV.

### Immunohistochemistry

The mice were anesthetized via an i.p. injection of avertin (250 mg/kg body weight, T48402, Sigma) and transcardially perfused with phosphate-buffered saline (PBS) followed by 4% formaldehyde. The brains were postfixed overnight at 4 °C in a 4% formaldehyde solution and then embedded in 5% agarose gel for sectioning (50-µm-thick coronal sections) with a vibratome (VT1000S, Leica). Sliced sections were blocked with 4% normal goat serum and 0.45% Triton X-100 in 0.1 M phosphate buffer at 4 °C for 1 h and then incubated with the following primary antibodies at 4 °C overnight: rabbit anti-GluN2B (AGC-003, Alomone Labs), rabbit anti-GluN2C (AGC-018, Alomone Labs), mouse anti-GluA2 (MAB397, Millipore) or mouse anti-GTP-Rac1 (26903, NewEast Bioscience) antibodies. Goat anti-rabbit DyLight 650-conjugated IgG (1:500, Bethyl Laboratories), goat anti-rabbit Alexa Fluor 568-conjugated IgG (1:500, Invitrogen) or goat anti-mouse DyLight 650-conjugated IgG (1:500, Bethyl Laboratories) were used as secondary antibodies. All the tissues were mounted on glass slides with UltraCruz mounting medium (Santa Cruz Biotechnology).

### Confocal imaging and laser capture microdissection

We used laser scanning confocal microscopes (LSM 510, Zeiss and FV3000, Olympus) with 40× or 60× objectives for cellular imaging of the colocalization of immunolabeled puncta on the dendrites. The quantitative analysis of immunoreactive puncta on the dendrites was performed with a color threshold (less than 80 was assumed to be the background noise intensity, and the threshold applied the green and red signals of pixels as colocalized) integrated with MetaMorph 7.7 (Molecular Devices).

Coronal brain sections (150 µm thick) were stained with 4′,6-diamidino-2-phenylindole to facilitate the alignment of the cell nucleus between the confocal and electron microscopy (EM) images. After confocal imaging, the sections were transferred to a laser capture microdissection system (Zeiss, PALM MicroBeam) for laser etching of the confocal imaged region. The etched sections were trimmed and stored in 2% paraformaldehyde and 2.5% glutaraldehyde in 0.15 M cacodylate buffer at 4 °C.

### Serial block-face scanning EM

The brain slices imaged under a confocal microscope were washed with cold 0.15 M sodium cacodylate buffer and placed in cacodylate buffer containing 2% OsO_4_/1.5% potassium ferrocyanide for 1 h. The tissues were placed in 1% thiocarbohydrazide (Ted Pella) in ddH_2_O for 20 min and then reacted in 2% aqueous OsO_4_ for 30 min. Thereafter, the samples were en bloc stained with 1% uranyl acetate overnight and a lead aspartate solution for 30 min to enhance the contrast of the membranes, as described previously^25^. The tissues were dehydrated using an ascending series of ethanol (50%, 70%, 90%, 95% and 100%) and placed in ice-cold dry acetone. The tissues were then gradually equilibrated with Epon 812 resin (Electron Microscopy Sciences) by placing them in a mixture of resin and acetone. The samples were embedded in fresh Epon 812 resin containing 7% carbon black (a kind gift from Dr. Nobuhiko Ohno), mounted on aluminum rivets, and cured in a 60 °C dry oven for 2 days to increase the conductivity of the samples.

Silver paste was applied to the sample to ground the carbon resin to the aluminum pin. The pin was then coated with 10 nm of gold‒palladium in a sputter coater (Q150RS) to further increase the conductivity. The samples were imaged with a Merlin field emission scanning electron microscope (Carl Zeiss) equipped with 3View2 technology (Gatan) and an OnPoint backscattered electron detector (Gatan). Photoshop software was used to overlay the low-magnification EM images atop the confocal images. The dendritic branching patterns of individual GFP-positive neurons were carefully considered to obtain optimal serial EM datasets, including their dendritic profiles. Serial EM images were acquired using an aperture of 30 μm, high vacuum, an acceleration voltage of 1.5 kV, an image size of 5,000 × 5,000 pixels, an image resolution (*XY* plane) of 11 nm, a dwell time of 3.5 µs and a section thickness of 50 nm. The serial images were converted to 8 bits and aligned with TrakEM2, an ImageJ plugin. eYFP-labeled neurons in confocal images were further confirmed with neurons in serial EM image stacks, and their dendritic profiles were manually segmented and three-dimensionally presented.

### Spine classification

The reconstructed dendritic structures were classified with three key parameters: spine length, head width and neck width. Spine length was manually determined by measuring the maximal length of the entry from the dendrites to the outermost part of the spine head. All the dendritic protrusions longer than 0.4 μm were considered for this analysis. For spine width, a line was drawn across the widest part of the dendritic protrusion, including the spine head. In accordance with these parameters, dendritic protrusions were classified into four classes as follows: (1) filopodia that had larger diameters than those of their necks and heads, necks similar in size to the head diameters, and over 3 μm of total length; (2) thin-shaped spines that were over 0.75 μm in total length and similar to their head and neck diameters; (3) stubby spines that had a neck diameter similar to or larger than the head diameter and whose total length was not longer than 0.75 μm; and (4) mushroom spines that had head diameters (over 0.35 μm) larger than those of their necks. These classes were then categorized into mature (stubby and mushroom) and immature (thin and filopodia) dendritic protrusions for macrolevel comparisons. The same parameters were also applied for the analysis of the confocal images in Fig. 2 and Supplementary Fig. 3.

### Postembedding immunogold EM

We performed transmission electron microscopy (TEM) for immuno-EM imaging with slight adaptations from the established protocol^26^. The methods used for transcranial perfusion and preparation of brain slices were identical to those used in the immunohistochemistry (IHC) protocols, except for the 200 μm thickness of the slices. A high-pressure freezing system (HPM 100, Leica) was used to freeze the tissues acutely while preserving the membrane and cellular components. Then, the sample tissues were incubated in acetone and embedded in Lowicryl HM20 resin (Electron Microscopy Sciences) at −45 °C for 2 days and subjected to ultraviolet polymerization for 1 day with EM AFS2 (Leica). Ultraviolet-polymerized blocks containing the NAcSh were sliced with an ultramicrotome (Leica). These slices were then placed on nickel grids (FCF200-Ni, Electron Microscopy Sciences). For immunostaining, the same primary antibodies that had been used in the IHC experiments (1:50 for GluN2B, GluN2C and GluA2 antibodies) paired with an anti-GFP antibody (1:50 for mouse monoclonal, sc-9996, Santa Cruz and rabbit polyclonal, LF-PA0046, AbFrontier) for eYFP detection were utilized. Subsequently, 18-nm gold particle-donkey anti-rabbit and 6-nm gold particle-donkey anti-mouse (1:50 for 711-215-152 and 715-195-150, Jackson ImmunoResearch) antibodies were used to label the rabbit (for GluN2B, C and GFP) or mouse (GluA2 and GFP) primary antibodies after blocking with 0.2% normal donkey serum in detergent-free PBS at 4 °C overnight. For the simultaneous staining of GFP, mCherry and GluN2B, we utilized the same mouse monoclonal anti-GFP antibody and rabbit anti-GluN2B antibody as previously described, with the exception of a rat monoclonal antibody specific for mCherry (1:25, M11217, Invitrogen). After overnight blocking at 4 °C with 0.2% normal goat serum in detergent-free phosphate buffer, we applied 18-nm gold-conjugated goat anti-mouse, 6-nm gold-conjugated goat anti-rat (1:25 for 115-215-146 and 112-195-14, respectively, Jackson ImmunoResearch) and 12-nm gold-conjugated goat anti-rabbit (1:25 for ab105298, Abcam) secondary antibodies. After the antibody application, we treated the sections with 1–2% uranyl acetate for 4 min and Reynolds solution for 2 min to obtain high-contrast images. Images were obtained using a transmission electron microscope (JEM-1011, JEOL) equipped with a charge-coupled device camera (ES1000W, 3611 × 2457 pixels, Gatan). We discarded spots showing inconsistent or distorted signals during focus adjustments and *X*–*Y* beam alignment from further analysis to ensure the accurate detection of gold particle signals and prevent false positives.

### Intracranial infusion of drugs or peptides

The mice were bilaterally administered 0.3 μl of either myristoylated Pep2m (50 μM, 3801, Tocris), PPDA (0.1 μM, 2530, Tocris) or vehicle (aCSF) via an injection cannula connected to a 10 μl Hamilton syringe. The microinfusion was conducted over 3 min at a rate of 0.1 μl/min using a microinfusion pump (Harvard Apparatus). After the injection, the mice were allowed 30 min to rest in their home cages before the start of the behavioral tests. The subject mice were perfused after the completion of the last behavioral tests, and the injection locations were verified. Any mice with incorrectly placed cannula tips were excluded from further analysis.

### Western blotting

The mice were anesthetized with avertin and transcardially perfused with sucrose cutting solution, and brain slices were generated with a vibratome (Leica) as previously described. NAc areas were separated by a steel borer (1.5 mm inner diameter), transferred to homogenization buffer containing 100 µl of RIPA buffer (Sigma) with 10 µl each of the protease inhibitor cocktail (Sigma) and phosphatase inhibitor cocktail (Sigma), and then sonicated. After sonication, the samples were centrifuged at 13,000 rpm for 15 min at 4 °C, and the supernatants were transferred to fresh tubes. Protein concentrations were measured using the Bradford protein assay (Bio-Rad Laboratories). The samples were prepared with 4× sample buffer (0.25 M Tris base (pH 6.8), 8% SDS, 40% glycerol, 0.002% bromophenol blue and 20% 2-mercaptoethanol) and heat denatured at 37 °C for 30 min. Twenty grams of protein per lane were subjected to electrophoresis on 8% bisacrylamide gels and transferred to polyvinylidene difluoride membranes. The membranes were blocked with 5% skim milk in PBST. After blocking, the membranes were incubated overnight at 4 °C with the following primary antibodies: mouse anti-GluN1 (1:1000 for 05-432, Millipore), rabbit anti-GluN2A (1:1000 for 07-632, Millipore), rabbit anti-GluN2B (1:1000 for AGC-003, Alomone Labs), mouse anti-GluN2B (1:500 for 610416, BD Transduction Lab), rabbit anti-GluN2C (1:1000 for AGC-018, Alomone Labs), rabbit anti-GluA1 (1:2000 for 13185, Cell Signaling Technology), mouse anti-GluA2 (1:2000 for MAB397, Millipore) or anti-β-actin (1:10,000 for A5441, Sigma). The membranes were washed three times for 10 min with PBST and incubated with horseradish-peroxidase-conjugated secondary IgGs (1:1000 for rabbit 170-6515, mouse 170-6516, Bio-Rad or 1:2000 for 7076s, Cell Signaling) for 1 h at room temperature. The signals from the membranes were detected with enhanced chemiluminescence (Immobilon Western, Millipore) using a charge-coupled device digital camera system (Amersham Imager 680, Cytiva). Proteins were quantified using ImageJ software, and all the data were normalized to those of the control proteins.

## Results

### Requirement of GluN2B for cocaine-induced silent synapses and addiction memory

It has been widely documented that physiological and structural changes in striatal circuits occur during extended abstinence periods (weeks to months) after the chronic administration of addictive drugs^19^. However, early forms of drug-evoked synaptic plasticity, if any, can permit metaplasticity for the late onset of adaptive physiological and behavioral changes with repetitive drug exposure. Thus, we monitored synaptic transmission and structural changes as early as several hours (<3 h) after the completion of the 5-day administration of cocaine.

**Fig. 1:**
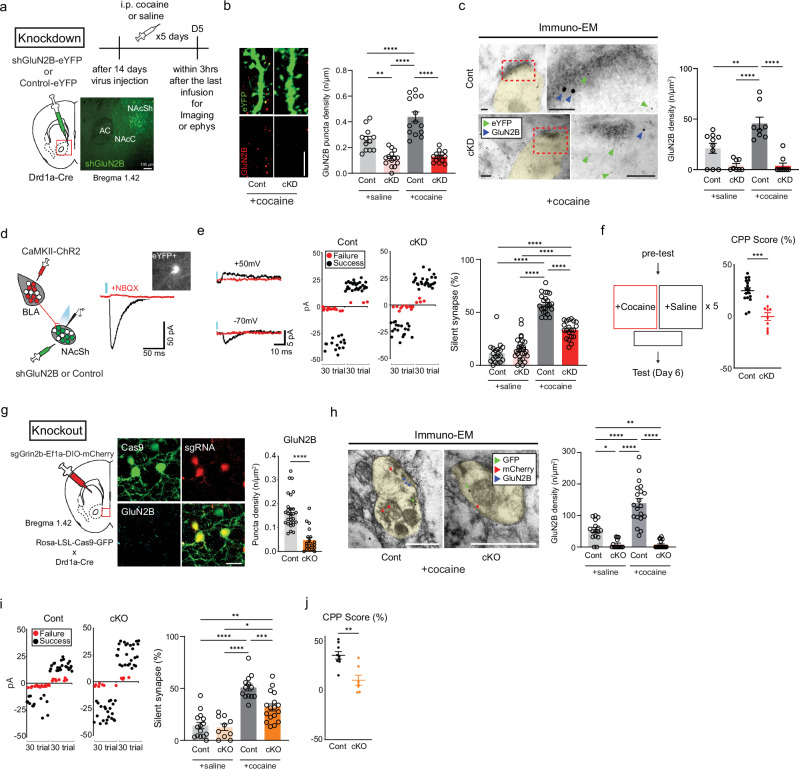
GluN2B is necessary for cocaine-induced silent synapses and addiction memory. **a**, Left: a schematic of shGluN2B or control virus injection into the NAcSh. Top: a timeline for the general experiments. Cocaine or saline was injected once every day (5 days). Bottom: the shGluN2B virus expressed in the NAcSh area. Scale bar, 100 μm. **b**, Left: representative images of GluN2B-positive puncta (red) in D1-MSN dendrites (green). Right: the density of GluN2B puncta after cocaine/saline administration was compared between mice that received either shGluN2B or the control virus (*n* = 12 cells for the saline/control group; *n* = 14 cells for the saline/cKD group; *n* = 14 cells for the cocaine/control group; *n* = 13 cells for the cocaine/cKD group). Scale bar, 5 μm. **c**, Left: immuno-EM images showing the subcellular localization of GluN2B (18 nm gold particles, blue arrows) and eYFP for labeling D1-MSNs (6 nm gold particles, green arrows). Right: the GluN2B particle density was compared between cKD D1-MSNs and control D1-MSNs after cocaine/saline administration (*n* = 10 cells for the saline/control group; *n* = 8 cells for the saline/cKD group; *n* = 8 cells for the cocaine/control group; *n* = 9 cells for the cocaine/cKD group). Scale bars, 100 nm. **d**, Left: a schematic of the optogenetic stimulation of the BLA-NAcSh pathway. Right: representative EPSC traces obtained after the optical stimulation (blue bar) of BLA glutamatergic inputs and an image obtained with (red) or without NBQX (black) showing cKD D1-MSNs (an inserted image). **e**, Left: sample traces of successful (black trace) and failure (red trace) responses to minimal optical stimulation of AMPAR-EPSCs (−70 mV) and NMDAR-EPSCs (+50 mV). Middle: representative plots of optical responses with minimal optical stimulation in the designated groups are shown (failure trials are shown as red dots, successful trials are shown as black dots). Right: the proportion of silent synapses was compared between cocaine/saline-treated mice that previously received either shGluN2B or the control virus (*n* = 19 cells for the saline/control group; *n* = 28 cells for the saline/cKD group; *n* = 23 cells for the cocaine/control group; *n* = 20 cells for the cocaine/cKD group). **f**, Left: a schematic of the conditional place preference test. Right: the quantified CPP scores in the cocaine chamber were compared between the mice that previously received either shGluN2B or the control virus (*n* = 17 control mice; *n* = 10 cKD mice). **g**, Left: a schematic of GluN2B cKO via CRISPR–Cas9. Middle: representative images of GluN2B (cyan) expression in D1-MSNs expressing Cas9–eGFP (green) and sgGrin2b–mCherry (red). Right: the GluN2B puncta density after cocaine administration was compared between mice that previously received either sgGrin2b or the control virus (*n* = 29 control cells; *n* = 19 cKO cells). Scale bar, 20 μm. **h**, Left: immuno-EM images showing the subcellular localization of GluN2B (12 nm gold particles, blue arrows), mCherry (6 nm gold particles, red arrows) and eYFP for labeling D1-MSNs (18 nm gold particles, green arrows). Right: the GluN2B particle density in the synaptic sites labeled with both eYFP and mCherry was compared between the cKO and control groups after saline/cocaine treatment (*n* = 16 cells for the saline/control group; *n* = 14 cells for the saline/cKO group; *n* = 20 cells for the cocaine/control group; *n* = 18 cells for the cocaine/cKO group). Scale bar, 100 nm. **i**, Left: representative plots of AMPAR- and NMDAR-EPSCs with minimal optical stimulation in the designated groups (failure trials, red dots; successful trials, black dots). Right: the proportion of silent synapses was compared between saline/cocaine-treated Cas9–eGFP mice that previously received either sgGrin2b or the control virus (*n* = 15 cells for the saline/control group; *n* = 10 cells for the saline/cKO group; *n* = 13 cells for the cocaine/control group; *n* = 17 cells for the cocaine/cKO group). **j**, The quantified CPP scores in the cocaine chamber were compared between the GluN2B cKO and control groups (*n* = 9 control mice; *n* = 7 cKO mice). The data are presented as the mean ± s.e.m. (error bars); *P < 0.05, **P < 0.01, ***P < 0.001, ****P < 0.0001 by Tukey post hoc analysis after two-way ANOVA, two-tailed unpaired *t*-test or the Mann‒Whitney test.

AMPAR-lacking silent synapses emerge mainly in D1-MSNs of the NAc after the repeated administration of cocaine^15^. Indeed, increased surface insertion of GluN2B-NMDARs results in increased numbers of silent synapses^5,15,16^, whereas acute depletion prevents the de novo formation of silent synapses^15^. We depleted GluN2B specifically in D1-MSNs of the NAcSh to explore the operational roles of silent synapses. This depletion was achieved through viral Cre-dependent expression of a shRNA targeting GluN2B (shGluN2B; Fig. 1a and Supplementary Fig. 1a)^9^, the efficacy of which was validated via western blotting (Supplementary Fig. 1b). Importantly, AAV containing shGluN2B led to an apparent reduction in GluN2B expression without any discernible effects on GluN2A or GluN1 expression (Supplementary Fig. 1b–d). Moreover, the ablation of GluN2B was confirmed through the quantification of GluN2B-positive puncta in D1-MSNs (Fig. 1b and Supplementary Fig. 1e) and an immuno-TEM-based ultrastructural analysis at the single-synapse level (Fig. 1c and Supplementary Fig. 1f).

GluN2B depletion impaired cocaine-induced synaptic plasticity and seeking behaviors^27^. We explored the physiological and behavioral impacts of GluN2B depletion, particularly in the pathway from the BLA to D1-MSNs, after 5 days of cocaine exposure. To gain optogenetic control of the BLA–NAc pathway, we conducted optical stimulation of the BLA via an infusion of an AAV encoding CamKIIα-ChR2-mCherry into the BLA of D1a-Cre mice that previously received the shGluN2B virus (Supplementary Fig. 1g). Optical stimulation of BLA fibers projecting to the NAc was sufficient to evoke AMPAR-mediated EPSCs from eYFP-labeled D1-MNS with a delay time of 19.63 ± 4.65 ms, which were abolished by NBQX (Fig. 1d). We assessed the proportion of silent synapses in D1-MSNs of mice that repeatedly received cocaine using a conventional method with minimal stimulation^27,28^. The photostimulation intensity was adjusted to the point where EPSC failure and success could be visually distinguished at −70 mV and +50 mV holding potentials, as previously described^29^. The calculated percentages of cocaine-induced silent synapses increased in both cocaine-treated groups but decreased in conditional knockdown (cKD) mice compared with those in the control group, whereas the same GluN2B depletion did not affect silent synapses in saline-treated mice (Fig. 1e and Supplementary Fig. 1h).

We used CPP to examine whether the shGluN2B-mediated reduction in the number of silent synapses could affect addiction memory^24^. The subject animals initially had no specific preference for any of the chambers before the cocaine injection (Supplementary Fig. 1i), but they subsequently preferred the chamber where cocaine was injected. However, D1a-Cre mice that received the shGluN2B virus bilaterally had no preference for any chamber (Fig. 1f), indicating that the shGluN2B-mediated reduction in the number of silent synapses interfered with the formation of addiction memory.

We excluded the possibility that any observed effects were due to off-target effects of the shRNA sequence used by also conditional knockout (cKO) of GluN2B in D1-MSNs using the CRISPR–Cas9 system, the efficacy of which was validated via western blotting (Supplementary Fig. 2a). When the AAV containing *sgGrin2b* was delivered to the NAc areas of LSL–Cas9–GFP mice that had been crossed D1a-Cre mice, we detected the colocalization of Cas9 and the sgRNA (Fig. 1g). The number of positive puncta and particle density of GluN2B were lower in cKO D1-MSNs than in control neurons (Fig. 1g, h and Supplementary Fig. 2b). Importantly, GluN2B cKO mice presented alterations in the proportion of silent synapses and cocaine-associated behaviors, which were almost identical to those of GluN2B cKD mice: a decrease in the proportion of silent synapses (Fig. 1i) and an impairment of CPP but normal appetitive memory for sucrose (Fig. 1j and Supplementary Fig. 2c). These data highlight the essential roles of GluN2B both in the generation or maintenance of silent synapses of D1-MSNs and in the expression of addiction memory.

### Cocaine-induced structural and physiological changes are facilitated by GluN2B ablation

**Fig. 2:**
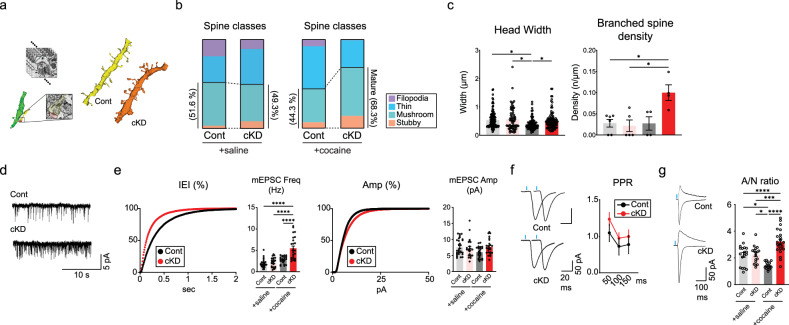
The structural and functional maturation of synapses is accelerated by GluN2B depletion. **a**, Left: a schematic of three-dimensional reconstructions from serial SEM images. Right: representative images of reconstructed dendrites from cocaine-treated cKD and control D1-MSNs. **b**, The proportion of morphologically categorized spine classes in control/shGluN2B virus-treated or cocaine-treated mice (saline/control group, *n* = 113 spines from 3 mice; saline/cKD group, *n* = 66 spines from 3 mice; cocaine/cKD group, *n* = 79 spines from 3 mice; cocaine/control group, *n* = 85 spines from 2 mice). **c**, Analysis of the average spine head width and density of branched spines compared between the cKD and control groups after saline/cocaine administration (head width, *n* = 130 units for the saline/control group; *n* = 66 units for the saline/cKD group; *n* = 85 units for the cocaine/control group, *n* = 95 units for the cocaine/cKD group; branched spine density, *n* = 6 units for the saline/control group; *n* = 5 units for the saline/cKD group; *n* = 4 units for the cocaine/control group, *n* = 4 units for the cocaine/cKD group). **d**, Sample traces of mEPSCs from D1-MSNs of cocaine-treated mice that previously received either shGluN2B or the control virus. **e**, Left: cumulative probability plot of the mEPSC interevent intervals (IEIs) after cocaine exposure and an inserted summary histogram of the quantified mEPSC frequency after saline/cocaine exposure (*n* = 30 cells for the saline/control group; *n* = 22 cells for the saline/cKD group; *n* = 25 cells each for the control and cKD groups after cocaine treatment). Right: cumulative probability plot of mEPSC amplitudes after cocaine exposure and a summary histogram of the quantified mEPSC amplitudes after saline/cocaine exposure (*n* = 30 cells for the saline/control group; *n* = 22 cells for the saline/cKD group; *n* = 25 cells each for the control and cKD groups after cocaine treatment). **f**, Left: sample traces of optically evoked EPSCs stimulated with a 50-ms interval in the D1-MSNs of saline-treated mice that previously received either shGluN2B or the control virus. Right: measurements of PPRs obtained with stimulation at 50-, 100- and 150-ms intervals in D1-MSNs from cocaine-treated mice that previously received either shGluN2B or the control virus are shown (*n* = 9 cells for the cKD groups; *n* = 11 cells for the control groups). **g**, Left: sample traces of AMPAR-EPSCs (at −70 mV) and NMDAR-EPSCs (+50 mV) in cocaine-treated D1-MSNs. Right: A/N ratios were compared between saline/cocaine-treated mice that previously received either shGluN2B or the control virus (*n* = 17 cells for the saline/cKD group; *n* = 18 for the saline/control group; *n* = 15 cells for the cocaine/control group; *n* = 22 cells for the cocaine/cKD group). The data are presented as the mean ± s.e.m. (error bars); *P < 0.05, ***P < 0.001, ****P < 0.0001 by Tukey’s post hoc analysis after two-way ANOVA.

In most cases, the functional modification and maturation of synapses are associated with structural changes in striatal neurons^7,30^. We resorted to EM to thoroughly investigate the structural features of D1-MSNs. Scanning electron microscopy (SEM) was utilized to examine subtle anatomical differences in dendritic structures between GluN2B-depleted D1-MSNs and control D1-MSNs. We conducted three-dimensional reconstructions of images obtained from SEM tomography, which elucidated complex wiring diagrams of D1-MSN dendrites (Fig. 2a). A subsequent examination of the reconstructed dendritic structures revealed differences in the morphological characteristics of the dendritic spines of D1-MSNs according to the individual groups: cocaine-treated cKD (cocaine/cKD), cocaine-treated eYFP-expressing control (cocaine/control), saline-treated cKD (saline/cKD) and saline-treated control (saline/control) groups. We categorized the dendritic protrusions into four classes based on their shape and appearance^31–33^: filopodia, thin spines, mushroom spines and stubby spines (detailed in the Methods). Our analysis revealed a substantial increase in the proportion of mushroom and stubby spines, indicative of mature synapses, in the cocaine/cKD group compared with the cocaine/control group, whereas substantially fewer filopodia and thin spines, normally considered immature synapses, were observed in the cocaine/cKD group (Fig. 2b). However, we failed to detect differences in general spine density within the treatment groups (Supplementary Fig. 3a) or in mature structures between the groups without cocaine treatment (Fig. 2b and Supplementary Fig. 3b). We also measured the dimensions of each type of protrusion. Cocaine treatment resulted in a substantial reduction in the head width of dendrites in the control group but not in the cKD group (Fig. 2c, left). In addition, we frequently detected increased numbers of branched spines in the cocaine/cKD group (Fig. 2c, right), whereas the volume of spines, the number of synaptic vesicles per unit perforated postsynaptic density and the area of the postsynaptic density did not differ between the groups (Supplementary Fig. 3c). Thus, the SEM-based anatomical analysis revealed that the ablation of GluN2B increased the number of morphologically defined mature synapses but did not affect the general spine density in several hours after repeated cocaine infusions.

We monitored mEPSCs to assess basal transmission in D1-MSNs. Consistent with our anatomical data, we detected substantial increases in the mEPSC frequency in the cocaine/cKD group compared with the cocaine/control group (Fig. 2d,e and Supplementary Fig. 3d–f). The paired-pulse ratios (PPRs) of evoked EPSCs did not differ among the groups, arguing against potential presynaptic alterations (Fig. 2f and Supplementary Fig. 3g). Meanwhile, the AMPAR/NMDAR-EPSC ratio (A/N ratio) was substantially increased in the cocaine/cKD group compared with the cocaine/control group (Fig. 2g and Supplementary Fig. 3h). These results support the notion that D1-MSNs lacking GluN2B undergo postsynaptic changes with increases in AMPAR-mediated components. Taken together, these physiological data suggest that the observed decrease in the proportion of silent synapses is due to facilitated maturation/unsilencing of silent synapses rather than an impairment in the generation of silent synapses per se, suggesting that silent synapses are precursors of AMPAR-containing functional synapses.

### GluN2B ablation-mediated recruitment of calcium-impermeable GluA2-AMPARs

**Fig. 3:**
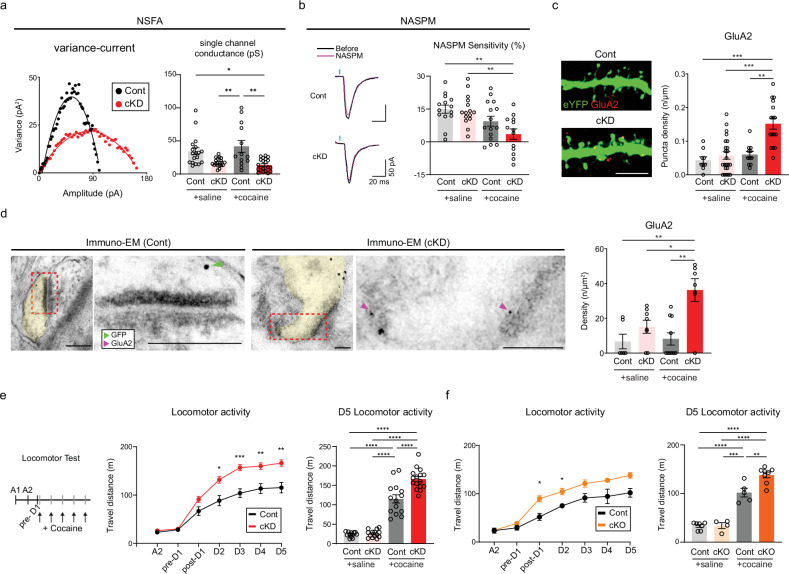
GluN2B ablation facilitates the recruitment of GluA2-AMPARs. **a**, Left: a variance‒current plot of cocaine-treated D1-MSNs (red, cKD groups; black, control groups). Right: the average single-channel conductance was compared between saline/cocaine-treated mice that previously received either shGluN2B or the control virus (*n* = 18 cells for the saline/control group; *n* = 17 cells for the saline/cKD group; *n* = 13 cells for the cocaine/control group; *n* = 21 cells for the cocaine/cKD group). **b**, Left: sample traces of evoked EPSCs before (black) and after (magenta) treatment with NASPM, an antagonist of GluA2-lacking AMPARs, in D1-MSNs from cocaine-treated mice that previously received either shGluN2B or the control virus. Right: the sensitivity to NASPM was compared between saline/cocaine-treated mice that previously received either shGluN2B or the control virus (*n* = 14 cells for the saline/control group; *n* = 15 cells for the saline/cKD group; *n* = 15 cells for the cocaine/control group; *n* = 13 cells for the cocaine/cKD group). **c**, Left: representative images of GluA2-positive puncta (red) in cocaine-treated D1-MSN dendrites (green). Right: the quantification of GluA2 puncta on D1-MSN dendrites between saline/cocaine-treated mice that previously received either shGluN2B or the control virus is shown (*n* = 9 cells for the saline/control group; *n* = 23 cells for the saline/cKD group; *n* = 11 cells for the cocaine/control group; *n* = 16 cells for the cocaine/cKD group). Scale bar, 5 µm. **d**, Left: immuno-EM images of the subcellular localization of GluA2 (6 nm gold particles, magenta arrows) and eYFP for the labeling of D1-MSNs (18 nm gold particles, green arrows). Right: GluA2 particles present within the synaptic areas were compared between saline/cocaine-treated mice that received either shGluN2B or the control virus (*n* = 6 cells for the saline/control group; *n* = 8 cells for the saline/cKD group; *n* = 10 cells for the cocaine/control group; *n* = 7 cells for the cocaine/cKD group). Scale bars, 100 nm. **e**, Left: experimental timeline for the measurement of cocaine-induced locomotor activity. Locomotor activity was monitored upon cocaine infusion on a daily basis between groups. Middle: cocaine-induced locomotion was compared between the cKD (red, *n* = 16 mice) and control (black, *n* = 14 mice) groups. Right: locomotor activity at D5 was compared between saline/cocaine-treated mice that previously received either shGluN2B or the control virus (*n* = 12 mice for the saline/control group; *n* = 14 mice for the saline/cKD group; *n* = 14 mice for the cocaine/control group, *n* = 16 mice for the cocaine/cKD group). **f**, Left: cocaine-induced locomotion was compared between the cKO (orange, *n* = 8 mice) and control (black, *n* = 5 mice) groups. Right: locomotor activity at D5 was compared between saline/cocaine-treated mice that previously received either shGluN2B or the control virus (*n* = 7 mice for the saline/control group; *n* = 4 mice for the saline/cKD group; *n* = 5 mice for the cocaine/control group, *n* = 8 mice for the cocaine/cKD group). The data are presented as the mean ± s.e.m. (error bars); *P < 0.05, **P < 0.01, ***P < 0.001, ****P < 0.0001 according to Tukey’s post hoc analysis after two-way ANOVA or the two-tailed Mann‒Whitney test.

GluN2B suppression was shown to increase the surface expression of AMPARs in hippocampal neurons in vitro^34^. If this process also occurred in NAc D1-MSNs, the decreased proportion of silent synapses and increased occurrence of functional types of synapses that we observed could be attributed to the premature unsilencing of silent synapses due to the promoted incorporation of AMPARs. It has been widely believed that recruitment of calcium-permeable (CP) AMPARs could mediate the maturation of silent synapses in D1-MSNs after cocaine exposure and underlie the incubation of addiction memory and craving traits^5,16,35^. However, we detected no rectification of AMPAR-EPSCs that CP-AMPARs normally displayed at depolarized holding potentials^36^ (Supplementary Fig. 4a,b). These results are seemingly consistent with previous reports showing that the rectification of AMPAR-EPSCs occurred during an extended abstinence period (>7 days) after repeated cocaine infusions^35,37^. Given that our anatomical and physiological data suggest precocious spine maturation of D1-MSNs in cocaine/cKD mice, the absence of AMPAR-EPSC rectification could be due to the facilitated recruitment of other types of AMPARs rather than CP-AMPARs. To this end, we took advantage of a NSFA of optically evoked AMPAR-EPSCs^38^ to approximate the single-channel conductance of individual AMPARs on D1-MSNs. Our NSFA results indicated that the single-channel conductance in the cocaine/cKD group was substantially lower than that in the cocaine/control group (Fig. 3a and Supplementary Fig. 4c), further arguing against the incorporation of CP-AMPARs^36^. We also perfused NASPM (200 μM), a selective antagonist for CP-AMPARs^39^, but we failed to detect any difference in NASPM sensitivity between the cKD and control groups, regardless of cocaine treatment (Fig. 3b and Supplementary Fig. 4d). These data provide experimental evidence that GluN2B ablation facilitates the recruitment of calcium-impermeable AMPARs (CI-AMPARs), but not CP-AMPARs, to D1-MSNs.

Given the lack of sensitivity changes to NASPM between the cKD and control groups, as well as the lack of rectification of AMPAR-EPSCs, GluA2-containing CI-AMPARs may be prime candidates for preferential synaptic recruitment^36^. To verify the incorporation of GluA2-AMPARs, we performed IHC and immuno-EM using an antibody against GluA2. Our IHC analysis revealed the apparent enrichment of GluA2-positive puncta in the dendrites of D1-MSNs from cocaine/cKD mice but not in those from the other groups (Fig. 3c and Supplementary Fig. 4e). Immuno-EM also revealed an elevated density of GluA2-positive particles within the synaptic areas in the cocaine/cKD group (Fig. 3d and Supplementary Fig. 4f). Thus, GluA2-AMPARs are likely to be prematurely recruited to D1-MSNs in cKD mice as early as several hours after exposure to cocaine, which accounts for the precocious maturation of silent synapses in D1-MSNs from cocaine/cKD mice.

Although it was well known that CP-AMPAR incorporation causes behavioral sensitization to cocaine^40^, whether CI-AMPARs play similar roles remains unknown. To explore behavioral impacts that the increased incorporation of GluA2-containing CI-AMPARs produced, we compared the cocaine-induced locomotor activity of cKD and cKO mice with that of the other groups. We observed substantial augmentation in locomotion activity to cocaine injections, namely hyperbehavioral sensitization, in both the cKD and cKO mice, whereas the control groups exhibited typical behavioral sensitization (Fig. 3e, f and Supplementary Fig. 4g,h). Collectively, the recruitment of GluA2-AMPARs to D1-MSNs appears to contribute to the premature unsilencing of silent synapses and thereby heightened behavioral sensitization to cocaine exposure.

### Cocaine-induced responses are restored by the blockade of GluA2-AMPAR trafficking

**Fig. 4:**
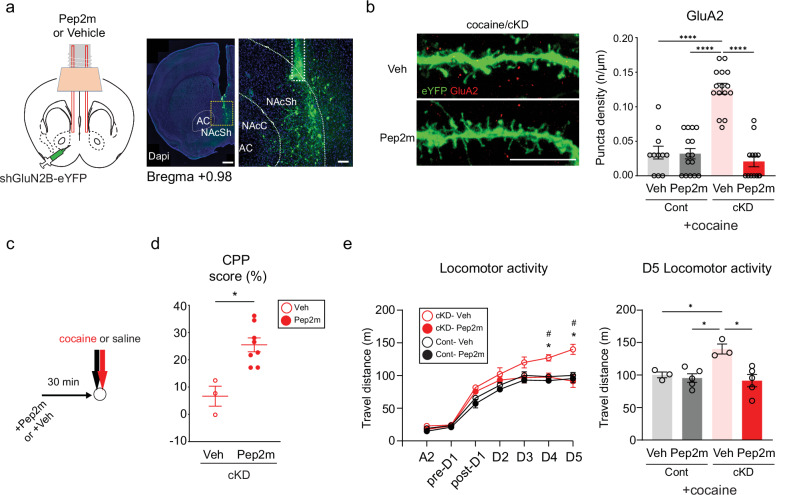
GluN2B depletion impairs addiction memory and increases behavioral sensitization via GluA2-AMPAR trafficking. **a**, Left: a schematic of Pep2m treatment. Right: representative images show the tips of guide cannulas (dashed line) in the NAcSh. Scale bars, 500 μm and 100 μm. **b**, Left: representative images of GluA2-positive puncta (red) in the dendrites of D1-MSNs (green) from cocaine/cKD mice. Scale bar, 10 µm. Right: the GluA2 puncta density in eYFG-labeled dendrites was compared between GluN2B cKD mice in which either Pep2m or the vehicle was infused into NAcSh areas (*n* = 11 cells for the cocaine/control + vehicle group; *n* = 15 cells for the cocaine/control + Pep2m group; *n* = 15 cells for the cocaine/cKD + vehicle group*; n* = 13 cells for the cocaine/cKD + Pep2m group). **c**, The experimental timeline for behavioral assessments after the Pep2m or vehicle infusion. **d**, Quantified CPP scores in the cocaine chamber after Pep2m or vehicle treatment were compared in mice that previously received the shGluN2B virus (*n* = 3 mice for the vehicle group; *n* = 8 mice for the Pep2m group). **e**, Left: the locomotor activity of control/GluN2B cKD mice was compared upon daily cocaine infusion after the Pep2m or vehicle treatment (*n* = 3 cells for the cocaine/control + vehicle group; *n* = 5 cells for the cocaine/control + Pep2m group; *n* = 3 cells for the cocaine/cKD + vehicle group; *n* = 5 cells for the cocaine/cKD + Pep2m group). Right: a comparison of D5 locomotor activity between cocaine-treated mice that previously received either shGluN2B or the control virus. The data are presented as the mean ± s.e.m. (error bars); ^#^*P* < 0.05, *P < 0.05, ****P < 0.0001, as determined by Tukey’s post hoc analysis after two-way ANOVA or the two-tailed Mann‒Whitney test.

Pep2m interferes with the interaction between the C-terminus of GluA2 and the *N*-ethylmaleimide-sensitive fusion protein, which can disrupt the trafficking of GluA2-AMPARs and reduce their surface expression^41,42^. We used a myristoylated form of Pep2m, a membrane-permeable peptide inhibitor, to examine the contribution of GluA2-AMPAR trafficking to the physiological and behavioral changes that we detected. The microinfusion of Pep2m into the NAcSh (Fig. 4a) effectively reduced the surface expression of GluA2 in D1-MSNs in the cocaine/cKD group but not in the cocaine/control group (Fig. 4b and Supplementary Fig. 5a).

We examined whether impaired trafficking of GluA2-AMPARs has any effect on the cocaine-induced behaviors of cKD mice. After the cKD mice received either Pep2m or vehicle bilaterally 30 min before each cocaine infusion (Fig. 4c), the CPP scores (the difference in time spent between the two chambers) were compared between the saline- and Pep2m-treated cKD mice (Fig. 4d). Compared with vehicle, Pep2m effectively reduced the locomotor activity of cKD mice but did not affect cocaine-induced locomotor changes in control mice (Fig. 4e). Taken together, the increased incorporation of GluA2-AMPARs probably underlies the abnormal behaviors in response to cocaine that we observed in cKD mice.

### Trafficking of GluA2-AMPARs is controlled by Rac1 activity

**Fig. 5:**
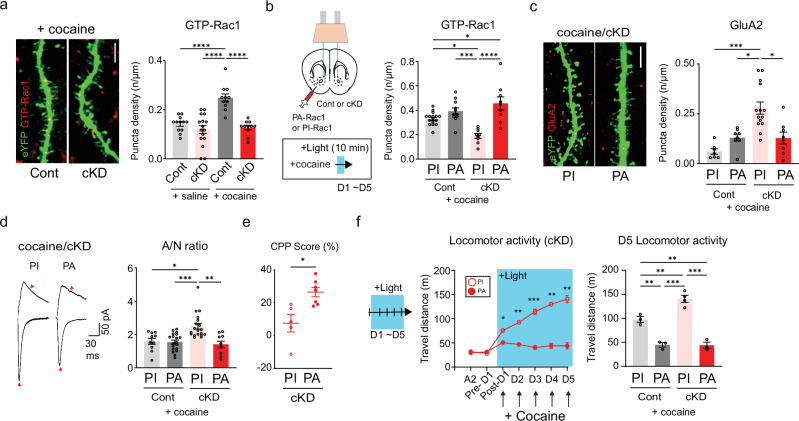
Rac1 controls the trafficking of GluA2-AMPARs. **a**, Left: representative confocal images of active Rac1-positive puncta (GTP-Rac1, red) in cocaine-treated D1-MSNs (green). Scale bar, 5 μm. Right: the density of GTP-Rac1-positive puncta after saline/cocaine administration was compared between mice that previously received either shGluN2B or the control virus (*n* = 12 cells for the saline/control group; *n* = 16 cells for the saline/cKD group; *n* = 11 cells for the cocaine/control group; *n* = 11 cells for the cocaine/cKD group). **b**, Left: experimental schematic for the photoillumination-triggered modulation of Rac1 activity. Right: the GTP-Rac1 punctum density after photoillumination and cocaine treatment was compared between control/cKD mice that previously received the PA-Racl or PI-Rac1 virus (*n* = 17 control/PI-Rac1 cells; *n* = 11 control/PA-Rac1 cells; *n* = 9 cKD/PI-Rac1 cells; n = 8 cKD/PA-Rac1 cells). **c**, Left: representative confocal images of active GluA2-positive puncta (red) on the dendrites of cocaine/cKD D1-MSNs (green) expressing PA- or PI-Rac1. Scale bar, 5 μm. Right: the GluA2-immunolabeled puncta quantified after photoillumination were compared between cocaine-treated control/cKD mice that previously received either the PA-Rac1 or PI-Rac1 virus (*n* = 7 control/PI-Rac1 cells; *n* = 8 control/PA-Rac1 cells; *n* = 14 cKD/PI-Rac1 cells; *n* = 8 cKD/PA-Rac1 cells). **d**, Left: sample traces of AMPAR- and NMDAR-EPSCs in cocaine/cKD D1-MSNs after photoillumination of PI or PA-Rac1. Right: A/N ratios were compared after photoillumination between cocaine-treated control/cKD mice that received either the PA-Rac1 or PI-Rac1 virus (*n* = 10 control/PI-Rac1 cells; *n* = 19 control/PA-Rac1 cells; *n* = 18 cKD/PI-Rac1 cells; *n* = 10 cKD/PA-Rac1 cells). **e**, Quantified CPP scores of the cocaine-paired chamber were compared after photoillumination between cKD mice that received either the PA-Rac1 or PI-Rac1 virus (*n* = 5 mice for the PI-Rac1 group; *n* = 7 mice for the PA-Rac1 group). **f**, Left: a photoillumination timeline for the measurement of locomotor activity. Light was delivered during and after cocaine infusions. Cocaine-induced locomotor activity was compared in GluN2B cKD animals that received either the PA-Rac1 or PI-Rac1 virus (*n* = 3 mice for the control group, *n* = 3 mice for the cKD/PA-Rac1 group; *n* = 4 mice for the cKD/PI-Rac1 group). Right: a comparison of D5 locomotor activity between cocaine-treated mice that previously received either shGluN2B or the control virus. The data are presented as the mean ± s.e.m. (error bars); *P < 0.05, **P < 0.01, ***P < 0.001, ****P < 0.0001 according to the two-tailed Mann‒Whitney or Tukey’s post hoc analysis after two-way ANOVA.

Synaptic and structural plasticity relies on actin dynamics within synaptic structures^43^. Structural and behavioral alterations can be mediated or controlled by Rac1-dependent actin remodeling^44–46^. Thus, we assessed Rac1 activity in each group of mice through immunolabeling for active GTP-bound Rac1^46^. Interestingly, D1-MSNs from cocaine/cKD mice presented lower Rac1 activity than those from the other groups (Fig. 5a and Supplementary Fig. 5b). We also attempted to manipulate Rac1 activity optically to examine its role in cocaine-induced physiological and behavioral changes using light-activatable Rac1: PA-Rac1 and PI-Rac1 as a negative control^21^. After DIO-AAV encoding either PA-Rac1 or PI-Rac1 was microinfused into D1a-Cre mice, the expression and functionality of transduced PA-Rac1 in eYFP-labeled D1-MSNs were confirmed by an increase in the density of GTP-Rac1 puncta after 473-nm light stimulation (Fig. 5b and Supplementary Fig. 5c). When Rac1 was optically stimulated, the expression of GluA2 decreased in D1-MSNs from cocaine/cKD mice compared with those from PI-Rac1 mice (Fig. 5c and Supplementary Fig. 5d). This finding suggested that the physiological and behavioral abnormalities caused by GluN2B ablation could be alleviated by Rac1 activity. Consistent with this notion, A/N ratios obtained from cocaine/cKD mice were normalized by the stimulation of Rac1 (Fig. 5d and Supplementary Fig. 5e), indicating that heightened activity of Rac1 was sufficient to resume synaptic transmission, potentially by impeding the aberrantly increased trafficking of GluA2-AMPARs in the dendrites of D1-MSNs of cKD mice. We also examined whether the activation of Rac1 mitigated the abnormal behaviors that were concurrent with the premature incorporation of GluA2-AMPARs. Stimulation of Rac1 restored the CPP to the cocaine-paired chamber in PA-expressing cKD mice, whereas we still detected impaired CPP in PI-expressing cKD mice (Fig. 5e). Furthermore, Rac1 activation throughout locomotion tests precluded any behavioral sensitization in cKD or control mice (Fig. 5f and Supplementary Fig. 5f). Overall, these results underscore that Rac1 is a critical regulator that can restrain the incorporation of GluA2-AMPARs and abnormal behaviors upon cocaine exposure, which are instigated by GluN2B ablation.

### GluN2B ablation triggers homeostatic switching of NMDAR subunits

**Fig. 6:**
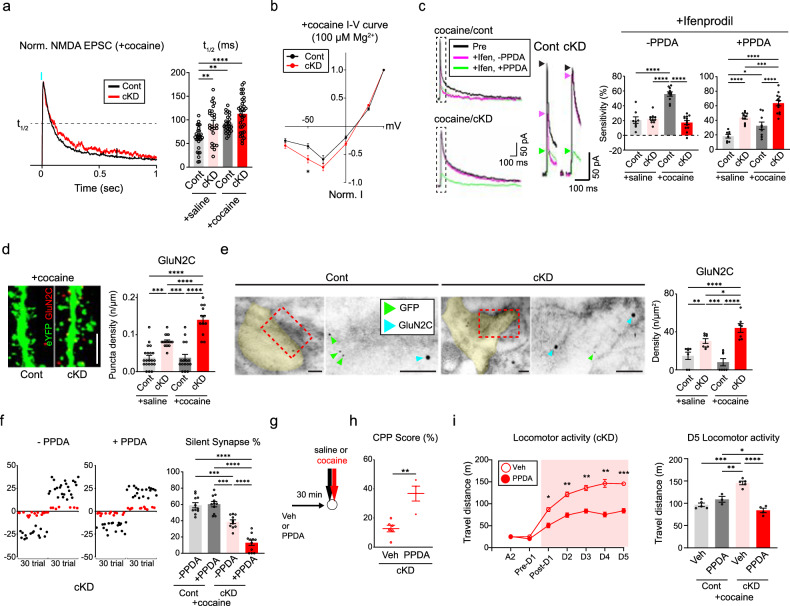
Homeostatic enrichment of GluN2C-NMDARs in the absence of GluN2B. **a**, Left: representative traces of normalized NMDAR-EPSCs (red traces for the cKD group; black traces for the control group). Right: the average NMDAR-EPSCs from D1-MSNs were compared between saline/cocaine-treated mice that received either the GluN2B cKD or control virus (*n* = 28 cells for the saline/control group; *n* = 25 cells for the saline/cKD group; *n* = 25 cells for the cocaine/control group; *n* = 33 cells for the cocaine/cKD group). **b**, *I*‒*V* curves of normalized NMDAR-EPSCs in the presence of 100 µM Mg^2+^ from D1-MSNs in cocaine-treated mice (red, *n* = 18 cells for the cKD group; black, *n* = 15 cells for the control group). **c**, Left: average traces of NMDAR-EPSCs from cocaine-treated D1-MSNs exposed to a GluN2B antagonist (ifenprodil, magenta) and an additional GluN2C antagonist (PPDA, green) at a +50 mV holding potential in the presence of PTX and NBQX. Middle: enlarged traces of NMDA-EPSCs with arrowheads denoting their peaks. Right: the sensitivity of NMDAR-EPSCs to ifenprodil or PPDA was compared between saline/cocaine-treated mice that previously received shGluN2B or the control virus (ifenprodil: *n* = 8 cells for the saline/control group, *n* = 10 cells for the cocaine/control group, *n* = 10 cells for the saline/cKD group, *n* = 13 cells for the cocaine/cKD group; PPDA: *n* = 8 cells for the saline/control group, *n* = 11 cells for the cocaine/control group, *n* = 9 cells for the saline/cKD group, *n* = 13 cells for the cocaine/cKD group). **d**, Left: representative confocal images of GluN2C-positive puncta (red) in cocaine-treated D1-MSNs (green). Scale bar, 5 μm. Right: the quantification of GluN2C puncta after saline/cocaine administration was compared between the mice that received either shGluN2B or the control virus (*n* = 21 for the saline/control group; *n* = 17 cells for the saline/cKD group; *n* = 16 cells for the cocaine/control group; *n* = 12 cells for the cocaine/cKD group). **e**, Left: immuno-EM images showing the subcellular localization of GluN2C (18 nm gold particles, cyan blue arrows) and eYFP for labeling D1-MSNs (6 nm gold particles, green arrows). Scale bar, 100 μm. Right: GluN2C particles present within the synaptic areas were compared between saline/cocaine-treated mice that previously received shGluN2B or the control virus (n = 7 cells for the saline/control group; *n* = 8 cells for the saline/cKD group; *n* = 7 cells for both the cocaine/cKD and cocaine/control groups). **f**, Left: representative plots of evoked EPSCs with minimal optical stimulation (failure trials are shown as red dots; successful trials are shown as black dots) and treatment without and with PPDA. Right: the proportion of silent synapses was compared before and after PPDA administration in saline/cocaine-treated mice that previously received the shGluN2B virus (*n* = 11 cells each). **g**, The experimental timeline of PPDA or vehicle treatment for behavioral assays. **h**, Quantified CPP scores in the cocaine-paired chamber were compared between PPDA- and vehicle-infused cKD mice (*n* = 4 mice for PPDA treatment; *n* = 6 mice for vehicle treatment). **i**, Left: locomotor activity was monitored daily upon cocaine infusion between PPDA- and vehicle-treated cKD mice (*n* = 4 mice for the PPDA groups; *n* = 5 mice for vehicle groups). Right: quantification of D5 locomotor activity between cocaine-treated mice that previously received either shGluN2B or the control virus (*n* = 5 mice for the vehicle/control group; *n* = 3 mice for the PPDA/control group; *n* = 5 mice for the vehicle/cKD group; *n* = 4 mice for the PPDA/cKD group). The data are presented as the mean ± s.e.m. (error bars); *P < 0.05, **P < 0.01, ***P < 0.001, ****P < 0.0001 according to two-tailed Mann‒Whitney or Tukey’s post hoc analysis after two-way ANOVA.

As indicated thus far, GluN2B ablation increased the abundance of GluA2-AMPARs in D1-MSNs of cocaine-treated mice much earlier than previously anticipated by inducing the premature unsilencing of silent synapses. However, we noted certain residual silent synapses even after the depletion or deletion of GluN2B (Fig. 1e,i), which raised the question of which NMDARs contributed to the presence of the remaining silent synapses. We compared optically evoked NMDAR-EPSCs at synapses from the BLA to D1-MSNs at +50 mV holding potentials in the presence of PTX and NBQX (Fig. 6a and Supplementary Fig. 6a). Because the decay kinetics of EPSCs normally depend on the composition of glutamate receptor subunits, we first measured the time taken to decay to the half peak amplitude (*T*_1/2_) rather than the weighted tau^15^. Although we depleted GluN2B, which displays slow decay kinetics^47^, we still observed comparable *T*_1/2_ values in cKD mice (Fig. 6a and Supplementary Fig. 6a). The decay kinetics represent the incorporation of other subunits of NMDARs that have similar or slightly slower decay kinetics than those of GluN2B-NMDARs.

In combination with two obligatory GluN1 subunits, other NMDAR subunits, such as GluN2A, GluN2C, GluN2D, GluN3A or GluN3B, can be incorporated into silent synapses when GluN2B is absent. To identify the subunit configuration of NMDARs in D1-MSNs lacking GluN2B, we perfused D1-MSNs with 100 µM Mg^2+^ and examined the voltage-dependent magnesium blockade of NMDAR-EPSCs. The resultant *I*–*V* curves indicated that NMDAR-EPSCs were less sensitive to Mg^2+^ in the cKD group than in the control group at a holding potential of −50 mV (Fig. 6b and Supplementary Fig. 6b). GluN2C/D-containing NMDARs (GluN2C-NMDARs) are less sensitive to Mg^2+^ than GluN2B-NMDARs are^47^. The decay kinetics of GluN2C-NMDARs are similar to those of GluN2B-NMDARs; GluN2D-NMDARs exhibit a much slower decay than do NMDARs containing GluN2B or GluN2C, whereas GluN2A-NMDARs have the fastest kinetics^47,48^. Therefore, our physiological data suggest that GluN2C-NMDARs are highly enriched in NAc D1-MSNs lacking GluN2B and thus are most likely to comprise silent synapses. Consistently, we observed increased expression of GluN2C, but not GluN2A, via western blotting of NAc tissues from cKD mice (Supplementary Fig. 6c).

We also performed a sequential antagonism test using specific inhibitors of either GluN2B or GluN2C. The application of ifenprodil (3 μM) led to substantial decreases in NMDAR-EPSC amplitudes in cocaine/control mice but not in those in the cocaine/cKD group (Fig. 6c), whereas ifenprodil sensitivity did not differ between groups without cocaine treatment (Supplementary Fig. 6d). When 0.1 μM PPDA, a well-characterized antagonist of GluN2C/D, was perfused into the same slices, the sensitivity to PPDA was much greater in cKD mice than in the control group (Fig. 6c and Supplementary Fig. 6d). We verified the incorporation of GluN2C-NMDARs into D1-MSNs by performing IHC and immuno-EM using an antibody against GluN2C. Our IHC results revealed the apparent enrichment of GluN2C-positive puncta in the dendrites of D1-MSNs from cKD mice but not in those from control mice, regardless of cocaine treatment (Fig. 6d and Supplementary Fig. 6e). Immuno-EM also revealed a greater density of GluN2C-positive particles in the synaptic areas of cKD mice than in those of control mice (Fig. 6e and Supplementary Fig. 6f). These physiological and anatomical results, together with the kinetics of the observed NMDAR-EPSCs, suggest that GluN2B can be replaced by GluN2C in D1-MSNs of mice that repeatedly received cocaine.

A reasonable speculation is that silent synapses could be installed by GluN2C-NMDARs when GluN2B is absent, leading to aberrant physiological and behavioral consequences. To explore this possibility, we infused the PPDA bilaterally into the NAcSh areas of cKD mice before each cocaine infusion. PPDA further reduced the proportion of remaining silent synapses in cocaine/cKD mice but did not affect it in the other groups (Fig. 6f and Supplementary Fig. 6g), indicating that GluN2C-NMDARs could contribute to the generation of silent synapses in D1-MSNs at least to some extent. Notably, the PPDA infusion restored typical cocaine-induced behaviors, including CPP and behavioral sensitization (Fig. 6g–i), similar to what we observed with Pep2m treatment and Rac1 activation. However, PPDA did not affect cocaine-induced locomotor activity in control mice (Supplementary Fig. 6h), suggesting a negligible contribution of GluN2C without cocaine injection and the specificity of PPDA for GluN2C-NMDARs.

## Discussion

GluN2B is a requisite component of silent synapses, which emerge during the abstinence period after the chronic administration of cocaine^15^. However, our knowledge of the functional roles and operational mechanisms of GluN2B-containing silent synapses remains limited because the *grin2b* gene can be lethal and developmentally effective^49^. We took advantage of virus-mediated Cre-dependent shRNA and CRISPR–Cas9 strategies to conditionally ablate GluN2B in a region- and cell-type-specific manner. Our multidisciplinary investigation involving a combination of electrophysiological recordings, cellular and ultrastructural analyses, and behavioral tests revealed that the ablation of GluN2B reduced the proportion of silent synapses upon cocaine exposure, most likely by facilitated recruitment of CI-AMPARs. The resultant AMPAR recruitment-mediated aberrant synaptic potentiation of D1-MSNs resulted in a deficit in CPP but an elevation of locomotor activity, leading to hyperbehavioral sensitization.

### Homeostatic switch in the NMDAR subunit composition of cocaine-induced silent synapses

Silent synapses in striatal D1-MSNs could play permissive roles in the development of specific responses that appear after repeated exposure to addictive drugs^5,15^. In particular, GluN2B is believed to be a key player in the generation or maintenance of silent synapses since either the depletion or antagonism of GluN2B reduces the number of cocaine-induced silent synapses^5,15^ and thereby affects behavioral sensitization and seeking behaviors^5,50^. However, how established silent synapses can be sustained after cocaine exposure and which mechanistic roles they play in the execution of addiction memory and addictive behaviors are largely unknown.

Despite the depletion or deletion of GluN2B, we could still detect the residual levels of silent synapses. Our physiological and anatomical analyses revealed that GluN2B was homeostatically replaced by GluN2C, which has a decay time course of NMDAR-EPSCs similar to that of GluN2B-NMDARs when GluN2B was ablated. It was previously demonstrated that the shift in NMDAR subunits is required for the development- and activity-dependent modification of synapses^51,52^. In fact, switching from GluN2B to GluN2C occurs in postnatal cerebellar granule cells^53–56^. However, how GluN2C is selected and recruited after 5 days of exposure to cocaine if it occurs in D1-MSNs lacking GluN2B remains unclear.

Given that GluN2C is rarely expressed in the striatal neurons of adult animals under normal conditions^57^, its expression in D1-MSNs would be upregulated, particularly in the absence of GluN2B. While whether GluN2C localizes to silent or functional synapses is uncertain, our findings suggest that GluN2C contributes primarily to the formation of silent synapses rather than their maturation into functional synapses. This hypothesis is supported by the sensitivity of residual silent synapses to the PPDA, which suggests that GluN2C is primarily targeted to nascent synapses. Consistently, PPDA reduced both the proportion of silent synapses and cocaine-induced locomotor sensitization in cKD mice.

However, we can surmise that GluN2A could also play certain roles in the homeostatic shifting of NMDA subunits, as previously suggested^17^. Future investigations of the state- and activity-dependent changes in the relative abundance of GluN2 subunits^47^ should provide novel insights into the mechanisms underlying the functional and behavioral outcomes driven by cocaine-induced silent synapses.

### Accelerated trafficking of AMPARs in the absence of GluN2B

As Pep2m can interfere with the trafficking of GluA2-AMPARs^41,42^, the increase in surface GluA2-AMPARs could be attributed to aberrantly facilitated trafficking of AMPARs into silent synapses, although we cannot completely exclude the possibility of increased production of GluA2 itself. Pep2m-induced normalization of CPP and locomotor activity in response to cocaine suggested that the enrichment of GluA2-AMPARs could account for the abnormal behaviors that mice lacking GluN2B exhibited upon cocaine exposure. These observations prompted us to hypothesize that a deficit in CPP and hyperbehavioral sensitization could result from the heightened excitatory drives that were induced by the premature recruitment of GluA2-AMPARs.

Structural alterations such as the generation of new synapses and the maturation of existing synapses rely on cytoskeletal dynamics^58^. The trafficking of AMPARs should also involve reorganization of the actin cytoskeleton, which allows the incorporation of AMPARs into silent synapses^43,58^. Rac1 is a well-known regulator that modulates actin remodeling in the dendrites and spines of neurons^45,46^, and when suppressed, it is able to drive the structural plasticity and functional maturation of MSN synapses upon cocaine exposure^3,46^. We also detected that the numbers of active GTP-bound Rac1 puncta decreased in D1-MSNs from cKD mice compared with those from control mice (Fig. 5a). However, acute stimulation of Rac1 normalized the CPP for cocaine but reduced locomotor activity of cKD mice and decreased the abundance of surface GluA2 in D1-MSNs, which differed from a previous report indicating that the same stimulation of Rac1 impaired both synaptic maturation and behavioral responses to cocaine^46^. Reduced GTP-Rac1 levels are linked to synaptic weakening and silent synapse formation during cocaine-seeking behavior^19^. The apparent discrepancies might be due to different experimental paradigms: D1-MSNs (in this study) versus general MSNs^19,46^; absence (in this study) versus presence of GluN2B^19,46^; noncontingent (in this study) versus contingent cocaine administration^19^; and synaptic strengthening (in this study) versus synaptic weakening stages of synaptic plasticity^19,46^. Kalirin 7, an upstream activator of Rac1, can be activated by GluN2B-NMDARs^59^, which controls the Rac1-mediated trafficking of AMPARs. Interestingly, KO mice deficient in Kalirin 7 exhibit hyperbehavioral sensitization and impaired CPP^60^, which was identical to what we observed in GluN2B cKD/cKO mice. Overall, Rac1 seems to be involved in the trafficking and specification of recruited AMPARs, although how the interplay between Rac1 and AMPARs could occur remains unclear.

### Precocious maturation of silent synapses with the recruitment of CI-AMPARs

The surface expression of GluA2 was substantially reduced by the perfusion of Pep2m and stimulation of Rac1, both of which interfered with the trafficking of GluA2-AMPARs. Because the maturation/unsilencing of silent synapses after exposure to cocaine is believed to be mediated by the incorporation of CP-AMPARs^35^, the facilitated incorporation of GluA2-containing CI-AMPARs was completely unexpected. Other types of AMPAR, rather than GluA2-lacking CP-AMPAR, could also unsilence cocaine-induced silent synapses, and the selection of AMPAR types could be managed by existing NMDAR types residing in those synapses.

It has been previously shown that GluN2B-NMDARs prevent the precocious maturation of developing synapses by blocking the premature trafficking of AMPARs^61^. The developmental capability of GluN2B-NMDARs to deter or slow down the trafficking of AMPARs would also be operative in cocaine-induced silent synapses, as if silent synapses that had been a developmental landmark in synapse formation became critical contributors to addiction memory and incubation^5,15,16^. Each subunit of NMDARs would have varied efficacies to prevent the recruitment of AMPARs: GluN2B deterred the incorporation of AMPARs most effectively, and GluN2A does so less effectively, whereas GluN2C does so least effectively or even attracts AMPARs. Elucidating these important issues requires a systematic analysis of the structural and biophysical features of various types of glutamate receptor.

In this work, we provide the first experimental evidence that GluN2B-NMDARs can specify the subunit composition of AMPARs that are subsequently incorporated into silent synapses. Therefore, Glu2B is critically required for the competency of silent synapses to allow the incorporation of CP-AMPARs over CI-AMPARs after cocaine exposure. As GluN2B can serve as a gateway to forthcoming outcomes of drug-induced silent synapses for addiction memory and related behaviors, transient and specific inhibitors of GluN2B, as well as suppressive genetochemical interventions, would be of great medical and therapeutic interest.

## Supplementary information

Supplementary Information
