# Supplementary material for: GluN2B-mediated regulation of silent synapses for receptor specification and addiction memory
Source: Exp Mol Med. 2025 Feb 10;57(2):436–49. doi: 10.1038/s12276-025-01399-z (PMC11873126; doi:10.1038/s12276-025-01399-z)
Supplement: Supplementary file 1 — Supplementary Information [file 12276_2025_1399_MOESM1_ESM.pdf]

Supplemental information

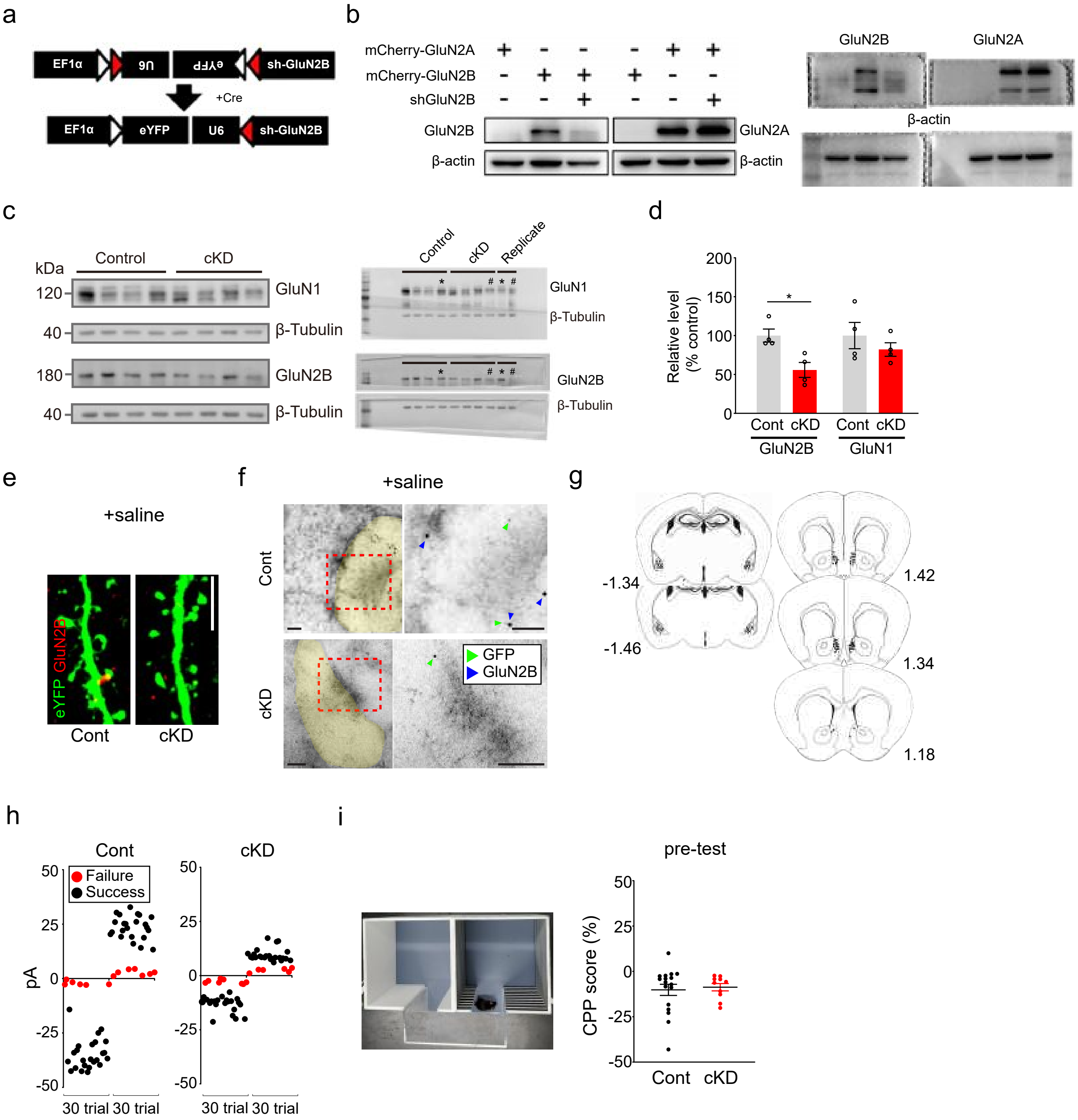

Kim et al., Supplementary Fig. 1

Supplementary Fig. 1. Validation of GluN2B depletion. **a,** A schematic diagram of the used construct and its expression for GluN2B knockdown. **b,** Western blot analysis of HEK293 cells expressing the designated constructs as well as β-actin as a loading control (left) and the original gel images (right). **c,** Western blot analysis of NAc tissues after viral infection (left) and the original gel images (right). **d,** A histogram of quantified protein amounts in designated conditions (n = 4 samples each for groups). **e,** Representative IHC images of GluN2B-positive puncta (red) in D1-MSNs dendrites (green). Scale bar = 5 μm. **f,** Immuno-EM images showing subcellular localization of GluN2B (18 nm gold particles, blue arrows) and eYFP for labeling of D1-MSNs (6 nm gold particles, green arrows, left). Scale bars = 100 nm. **g,** Verified location of microinjection sites in the NAc. Brain images (adapted from Paxinos and Watson, 2001)[62] corresponding to the NAc are overlapped, and injection sites are represented by black dots. Injector sites are located in brain slices at indicated distances (mm) from the bregma. **h,** Example plots of optical responses with minimal optical stimulation in the designated groups (failure trials in red dots; successful trials in black dots, left). **i,** An actual behavioral setting for CPP (left). CPP was tested before cocaine exposure (n = 10 mice for cKD; n = 17 mice for control, right). Data are represented as mean ± SEM (standard error of the mean); $^{*}p < 0.05$ by two-tailed Mann-Whitney.

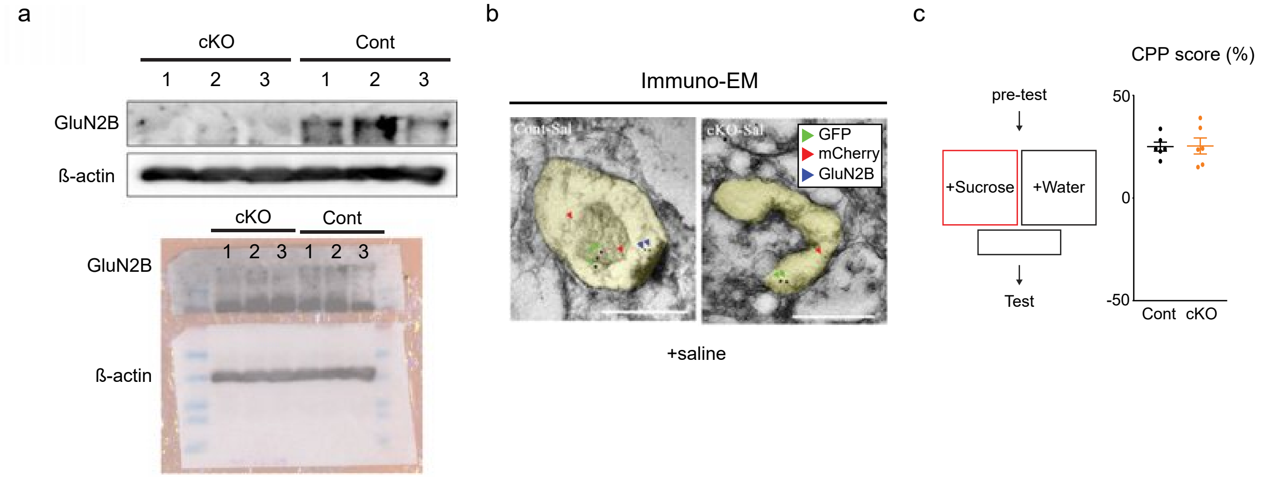

Kim et al., Supplementary Fig. 2.

Supplementary Fig. 2. Validation of GluN2B deletion. **a,** Western blot analysis of NAc tissues of mice that received either sgGrin2b or control virus (n = 3 samples each, top) and the original gel images (bottom). **b,** Immuno-EM images showing subcellular localization of GluN2B (18 nm gold particles, blue arrows) and eYFP for labeling of D1-MSNs (6 nm gold particles, green arrows, left). Scale bars = 100 nm. **c,** An experimental configurations for sucrose CPP (left). CPP scores in the sucrose-paired chamber are compared between cKO and control mice (n = 6 mice each for control and cKO groups, right). Data are represented as mean ± SEM (standard error of the mean).

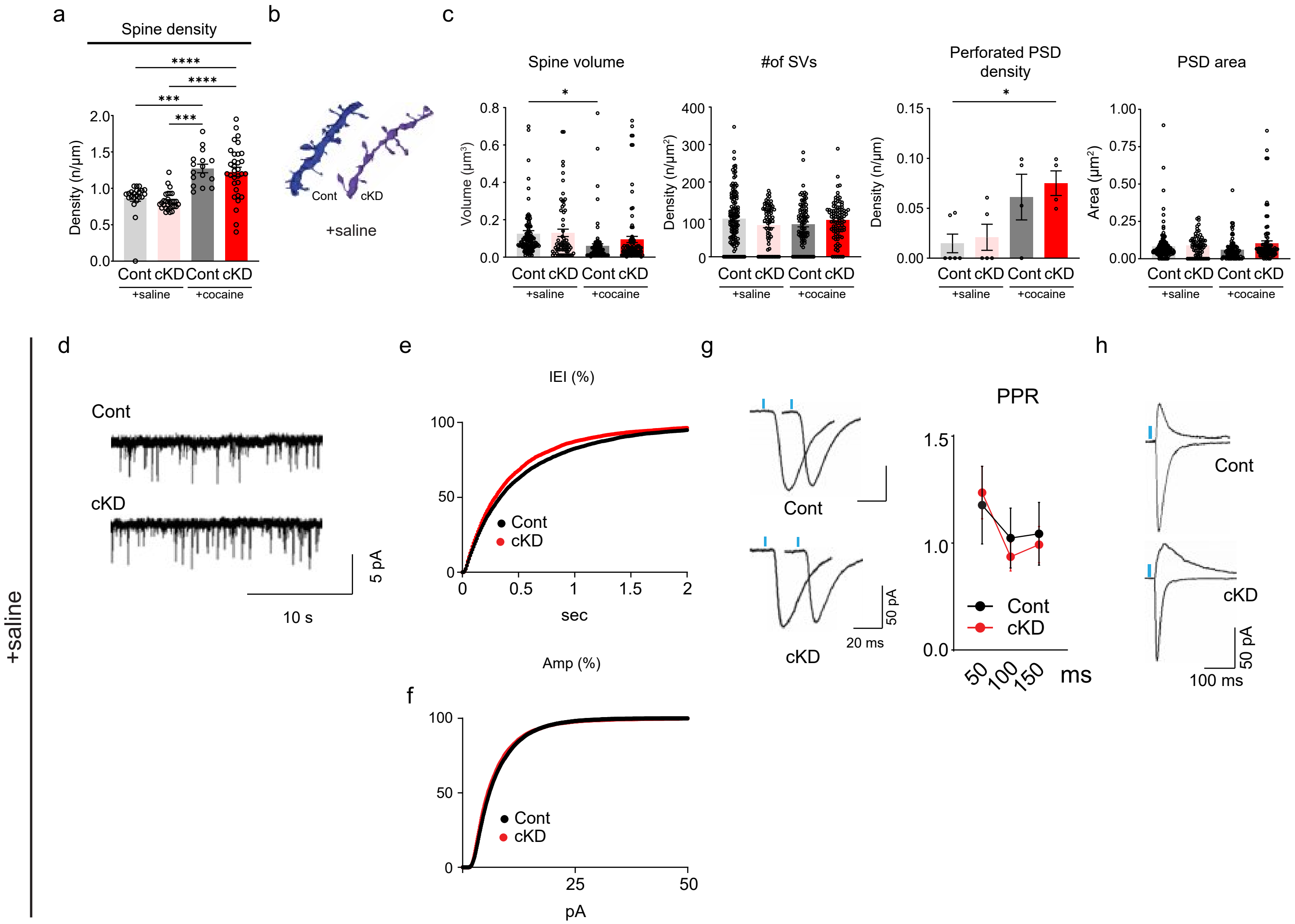

Kim et al., Supplementary Fig. 3

Supplementary Fig. 3 Structural and functional features of D1-MSNs in the absence of GluN2B. **a,** Spine density of D1-MSNs is compared between cocaine/saline treated mice that previously received either shGluN2B or control virus (n = 21 cells for saline/control; n = 29 cells for saline/cKD; n = 16 cells for cocaine/control n = 32 cells for cocaine/cKD). **b,** Representative illustrations of reconstructed dendrites and protrusions in saline-treated D1-MSNs **c,** Structural parameters (spine volume, # of SVs, perforated PSD density, PSD area) are compared between cocaine/saline-treated mice that previously received either shGluN2B or control virus (spine volume, # of SVs, PSD area: n = 130 units for saline/control; n = 66 units for saline/cKD; n = 85 units for cocaine/control; n = 95 units for cocaine/cKD, density of perforated PSD: n = 6 units for saline/control; n = 5 units for saline/cKD; n =4 units each for cocaine treated groups). **d,** Sample traces of mEPSCs from D1-MSNs of saline-treated animals that previously received either shGluN2B or control virus. **e,** A cumulative probability plot of mEPSC inter-event intervals (IEIs) after saline exposure. **f,** A cumulative probability plot of amplitude after saline exposure. **g,** Sample traces of optically-evoked EPSCs with stimulation of 50-ms interval in D1-MSNs of saline-treated mice that previously received either shGluN2B or control virus (left). Measurement of PPRs with stimulation of 50-, 100-, and 150-ms intervals in D1-MSNs of saline-treated mice that previously received either shGluN2B or control virus (n = 10 cells for control; n= 14 cells for cKD, right). **h,** Sample traces of optically-evoked AMPAR- (at −70 mV) and NMDAR-EPSCs (+50 mV) in D1-MSNs of saline-treated mice that previously received either shGluN2B or control virus. Data are represented as mean ± SEM (standard error of the mean). $^{*}p < 0.05$, $^{***}p < 0.001$, $^{****}p < 0.0001$ by Tukey *post hoc* analysis after two-way ANOVA.

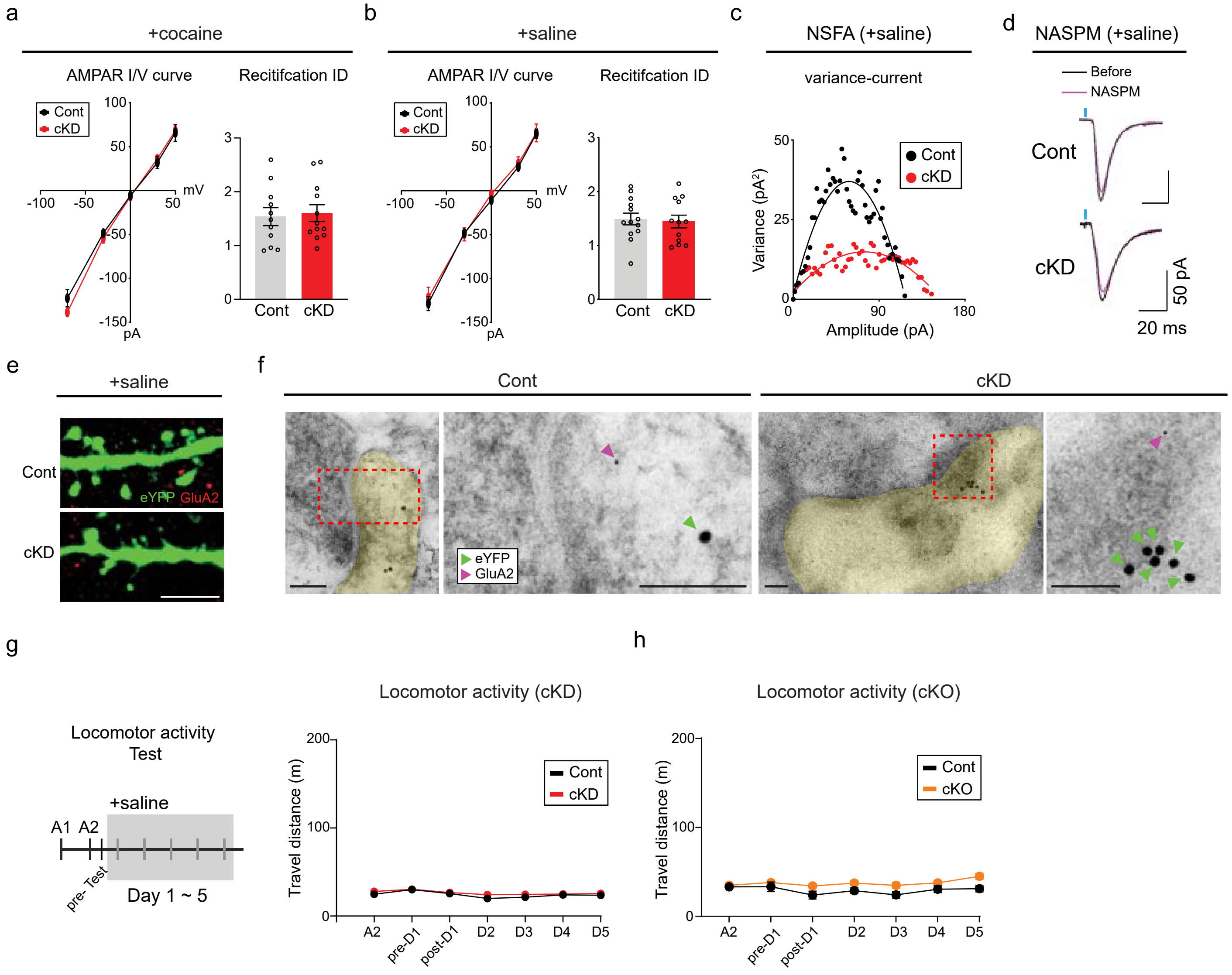

Kim et al., Supplementary Fig. 4.

Supplementary Fig. 4 Comparative analysis of AMPAR features. **a,** AMPAR I/V curves in cocaine-treated D1-MSNs (left). The rectification indices are compared between cocaine-treated mice that previously received either shGluN2B or control virus (n =11 cells for cocaine/control; n = 12 for cocaine/cKD, right). **b,** AMPAR I/V curves in saline-treated D1-MSNs (left). The rectification indices are compared between saline-treated mice that previously received either shGluN2B or control virus (n =13 cells for saline/control; n = 12 for saline/cKD, right). **c,** Representative variance-current plots for saline-treated D1-MSNs. **d,** Sample traces of EPSCs before (black) and after (magenta) NASPM treatment in saline-treated D1-MSNs. **e,** Representative IHC images for GluA2-positive puncta (red) on eYFP-labelled dendrites of saline-treated D1-MSNs (green). Scale bar = 5 μm. **f,** Representative immuno-EM images showing subcellular localization of GluA2 (6 nm gold particles, magenta arrows) and eYFP for labeling D1-MSNs (18 nm gold particles, green arrows). Scale bar = 100 nm. **g,** a schematic timeline for locomotion measurement (left). saline-induced locomotor activity is compared between mice that received either shGluN2B or control virus (n = 12 mice for control; n = 14 for cKD, right). **h,** Saline-induced locomotor activity is compared between Cas9-eGFP mice that received either sgGrin2b or the control virus (n = 5 mice each). Data are represented as mean ± SEM (standard error of the mean)

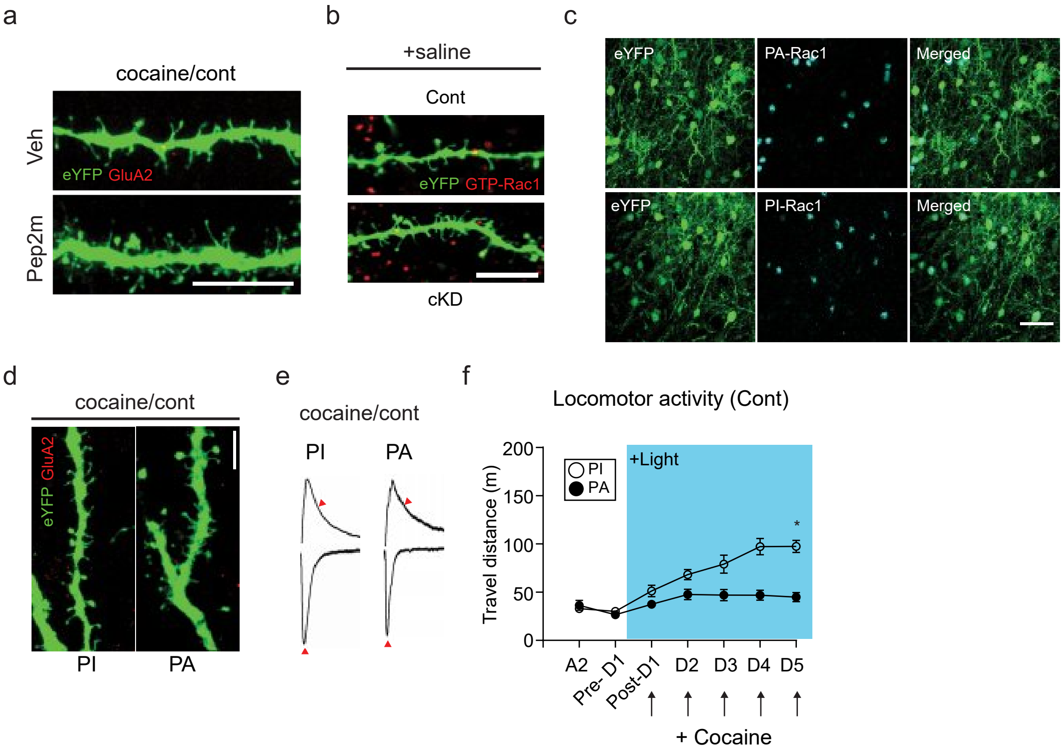

Kim et al., Supplementary Fig. 5

Supplementary Fig. 5. Rac1 mediates the regulation of GluA2 enrichment and cocaine-associated behaviors. **a,** Representative IHC images for GluA2-positive puncta (red) in dendrites of cocaine/control D1-MSNs (green, left). Scale bar = 5 μm. **b,** Representative IHC images of GTP-Rac1 positive puncta (red) in dendrites of saline-treated D1-MSNs (green, left). Scale bar = 5 μm. **c,** Representative fluorescence images for PA-Rac1 or PI-Rac1 expression (cyan, mcherry signals) in somas and dendrites of D1-MSNs (green, left). Scale bar = 50 μm. **d,** Representative IHC images of GluA2-positive puncta (red) on dendrites of cocaine/control D1-MSNs (green) expressing either PA- or PI-Rac1 (left). Scale bar = 5 μm. **e,** Example plots of EPSCs evoked with minimal optical stimulation in designated groups. The measurement points for AMPAR- (bottom) or NMDAR-EPSCs (top) are marked with red arrowheads. **f,** Cocaine-induced locomotor activity with photoillumination is compared between control mice that receive either PA-Rac1 or PI-Rac1 virus ($n = 3$ mice each). Data are represented as mean ± SEM (standard error of the mean); $^{*}p < 0.05$ by Tukey *post hoc* analysis after two-way ANOVA.

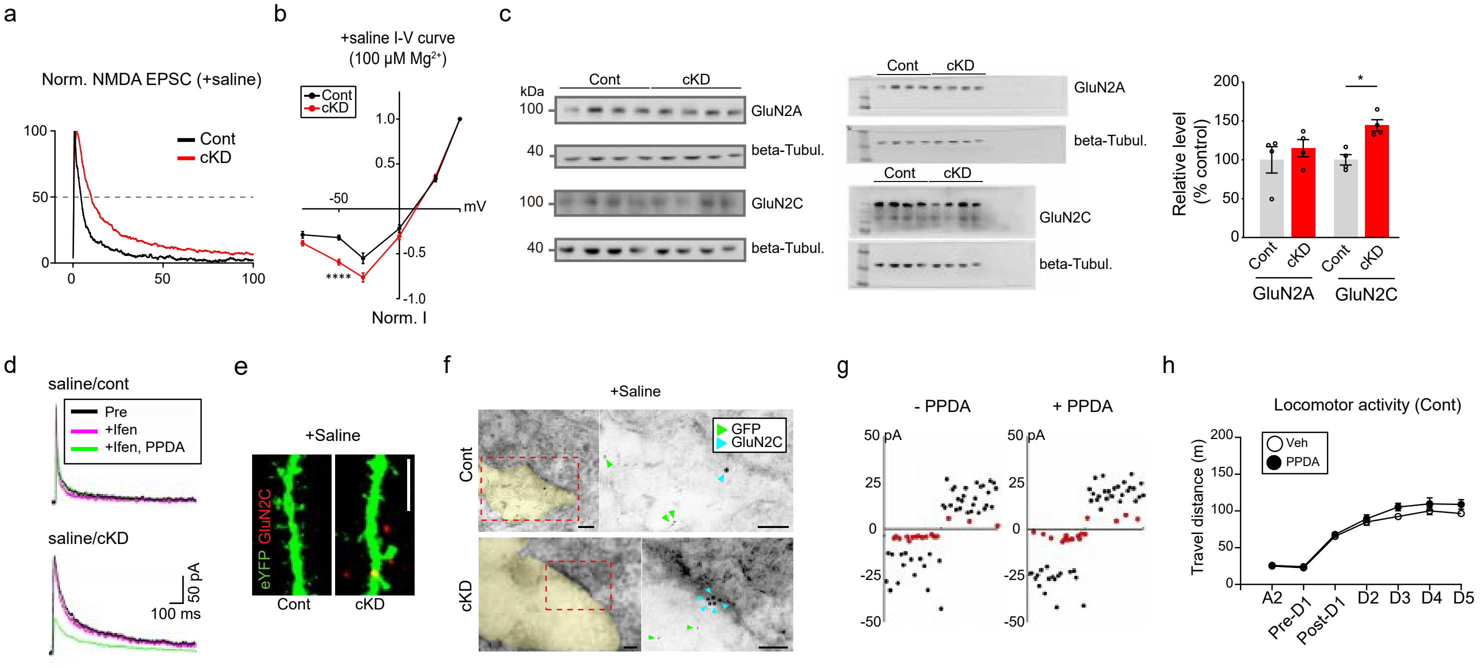

Kim et al., Supplementary Fig. 6

Supplementary Fig. 6. GluN2C contributes to silent synapses in the absence of GluN2B. **a,** Example traces of normalized NMDAR-EPSCs from saline-treated control (black) and cKD (red, D1-MSNs. **b,** I-V curves of normalized NMDAR-EPSCs under 100 μM of Mg^{2+} in saline-treated D1-MSNs (n = 14 cells each). **c,** Western blot analysis for GluN2A and GluN2C expression with biopsy samples of the NAc (left) and the original gel images (middle). Western blot data are quantified and compared between mice that receive either shGluN2B or control virus (n = 4 samples each, right). **d,** Example traces of NMDAR-EPSCs in saline-treated D1-MSNs with sequential application of a GluN2B antagonist (Ifenprodil, magenta) and a GluN2C/D antagonist (PPDA, green) at +50 mV holding potential. **e,** Representative IHC images of GluN2C-positive puncta (red) on dendrites of saline-treated D1-MSNs (green). **f,** Immuno-EM images showing subcellular localization of GluN2C (18 nm gold particles, blue arrows) and eYFP for labeling of D1-MSNs (6 nm gold particles, green arrows). **g,** Example plots of evoked EPSCs with minimal optical stimulation (failure trials in red dots; successful trials in black dots) without and with PPDA. **h,** Cocaine-induced locomotor activity is compared between control mice that were infused with either PPDA or vehicle (n = 3 mice for PPDA; n = 5 mice for vehicle). Data are represented as mean ± SEM (standard error of the mean); $^{*}p < 0.05$ by two-tailed Mann-Whitney test.

Reference

62 Paxinos, G., Franklin, K. B. J. & Franklin, K. B. J. *The mouse brain in stereotaxic coordinates*. 2nd edn, (Academic Press, 2001).
